# Quantitative Trait Loci Sequencing and Genetic Mapping Reveal Two Main Regulatory Genes for Stem Color in Wax Gourds

**DOI:** 10.3390/plants13131804

**Published:** 2024-06-29

**Authors:** Zhihao Chen, Peng Wang, Wenhui Bai, Yan Deng, Zhikui Cheng, Liwen Su, Lifeng Nong, Ting Liu, Wenrui Yang, Xiping Yang, Zhengguo Liu

**Affiliations:** College of Agriculture, Guangxi University, Nanning 530000, China; 2217391003@st.gxu.edu.cn (Z.C.); 20220135@gxu.edu.cn (P.W.); 2217391001@st.gxu.edu.cn (W.B.); 2217302002@st.gxu.edu.cn (Y.D.); 2217401017@st.gxu.edu.cn (Z.C.); 2317401015@st.gxu.edu.cn (L.S.); 2117302006@st.gxu.edu.cn (L.N.); 2117391028@st.gxu.edu.cn (T.L.); 2117391054@st.gxu.edu.cn (W.Y.); xipingyang@gxu.edu.cn (X.Y.)

**Keywords:** wax gourd, regulatory gene, stem color, gene mapping, sequencing, chlorophyll

## Abstract

Stem color is an important agronomic trait of wax gourds. However, its regulatory genes have not been identified. In this study, 105 inbred lines constructed from two parents (GX-71 and MY-1) were sequenced and quantitative trait loci sequencing was used to mine the genes that regulate stem color in wax gourds. The results identified two quantitative trait loci related to stem color, *qSC5* and *qSC12*, located on Chr05 (11,134,567–16,459,268) and Chr12 (74,618,168–75,712,335), respectively. The *qSC5* had a phenotypic variation rate of 36.9% and a maximum limit of detection of 16.9. And the *qSC12* had a phenotypic variation rate of 20.9%, and a maximum limit of detection of 11.2. *Bch05G003950* (named *BchAPRR2*) and *Bch12G020400* were identified as candidate genes involved in stem color regulation in wax gourds. The chlorophyll content and expression of *BchAPRR2* and *Bch12G020400* were significantly higher in green-stemmed wax gourds than in white-stemmed ones. Therefore, *BchAPRR2* and *Bch12G020400* were considered the main and secondary regulatory genes for wax gourd stem color, respectively. Finally, InDel markers closely linked to *BchAPRR2* were developed to validate the prediction of wax gourd stem color traits in 55 germplasm lines, with an accuracy of 81.8%. These findings lay the foundation for exploring the genetic regulation of wax gourd stem color and future research on wax gourd breeding.

## 1. Introduction

The wax gourd [*Benincasa hispida* (Thunb) Cogn. (2n = 2x = 24)] is an important crop of the Cucurbitaceae family. It is an annual herb that has been cultivated in China for ~1500 years. Owing to its nutritional and health benefits, wax gourd is a staple vegetable in China, especially in southern China [1,2].

Stems are important organs that can greatly affect crop yield; therefore, it is of great significance to explore stem-related traits. In 2022, Luo et al. [3] reported that plants with an overexpression of *BhSAUR60* exhibited wavy stems. However, the genes controlling wax gourd stem color have not been identified. In recent years, genes related to pigment traits in gourds have been revealed. In watermelon and melon, *CmAPRR2* is related to pigment accumulation in fruits [4]. In balsam pear, *APRR2* regulates stigma color and green stigma trait. It is located in the 13.87 kb region and has an exonic base insertion, which leads to structural changes in the encoded protein. [5]. *HG_GLEAN_10010973* is associated with the formation of green skin in bottle gourds [6]. In zucchini, the deletion of a 14 kb chromosomal fragment between CP4.1LG15g03360 and CP4.1LG15g0420 of the repeated locus on *APRR2* was reported to affect chlorophyll synthesis in stems, leading to the production of white-stem varieties of zucchini [7]. In wax gourd, *Bch05G003950* encodes a bicomponent response regulator-like protein, *Arabidopsis* pseudo-response regulator 2 (APRR2). Mutations of two bases in this gene lead to the formation of a premature termination codon and the inhibition of chlorophyll development and synthesis, resulting in the formation of white-skin varieties of wax gourds [8]. Huang et al. [9] reported that *Bch5G003950* is related to peel color in wax gourd. Thus, while many pigment-related genes in gourds have been mapped, few pigment-related genes for stems have been identified. The report of *Bch5G003950* in wax gourd peel color provides inspiration that *Bch5G003950* is a pigment-related gene that may be helpful in our studies of stem color.

Chlorophyll, carotenoids, and anthocyanins are important plant pigments. Chlorophyll (a and b), found in green tissues of plants, helps capture light energy. It is synthesized and located in chloroplasts [10,11]. Mutations in plant leaf color-related genes can lead to changes in chlorophyll and anthocyanins [12,13]. Thus, chlorophyll, as a key factor in photosynthesis, is important for improving crop yield. Therefore, researchers are committed to studying the synthesis and regulatory mechanisms of chlorophyll. For example, in one study, a key single nucleotide polymorphism mutation in *CmYGP* resulted in yellow–green plant traits in melons. *CmYGP* encodes a Golden2-like transcription factor that is highly expressed in green tissues. Virus-induced gene silencing further confirmed that CmYGP reduced the number of chloroplasts and chlorophyll content, resulting in the formation of yellow–green melon leaves and fruits [14]. Studies have shown that chlorophyll and auxins are intertwined in *Arabidopsis thaliana*; through ARF7-IAA14 mediation, auxin inhibits the chlorophyll synthesis gene in *A. thaliana* and subsequently chlorophyll accumulation. These findings provide new insights into the regulatory mechanisms of chlorophyll synthesis and accumulation [15]. In another study on tomatoes, the inhibition of *Slym1* promoted chlorophyll decomposition, which changed the leaf color [16]. This suggests that the downregulation of *SLMYB72* of the R2R3MYB subfamily, which regulates the metabolism of chlorophyll, carotenoids, and flavonoids, causes an uneven coloration in tomato fruit [17]. Several studies have shown that GLKs, TKN2, TKN4, and APRR2 are factors related to pigments [18,19,20] that regulate the production, differentiation, and function of chloroplasts. They play important roles in immune and stress responses and are important nodes in the regulatory network [21]. BEL1-LIKE HOMEODOMAIN4 can inhibit the expression of *TKN2*, which alters the chlorophyll content and chloroplast development in tomatoes [22]. Although research on pigment genes is diverse, studies on the stem color of cucurbit crops are lacking, and candidate genes regulating stem color need to be identified and analyzed.

Therefore, this study aimed to explore the mechanisms underlying the generation of plant pigments in stems. To elucidate the genes regulating stem color in wax gourds, we constructed a high-density genetic map (HDM) of wax gourds using quantitative trait loci (QTL) sequencing and resequencing data of a high-generation inbred line GX-71 (green stem), a high-generation inbred line MY-1 (white stem), and 105 RIL populations. 

## 2. Results

### 2.1. Phenotypic and Genetic Analyses of the Stem Color

**Figure 1:**
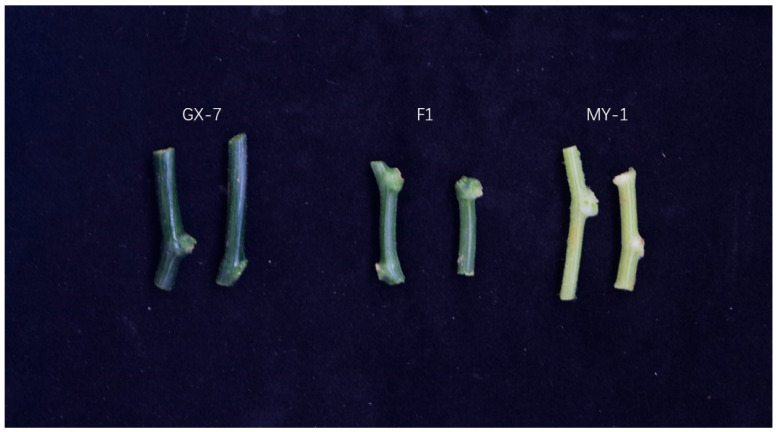
Phenotypic differences in stem color of the parents, GX-71 and MY-1, and the F_1_ generation.

The stem colors of the parents, GX-71 and MY-1, used in this study significantly differed. In the field, GX-71, MY-1, and the F_1_ generation had green, white, and light green stems, respectively (Figure 1); therefore, these gourds were selected for constructing populations used in the mining of stem color-related QTLs and candidate genes. To analyze the heritability of the stem color, an F_2_ population of 786 individuals was bred from GX-71 and MY-1. In the F_2_ population, 449 and 337 strains had green and white stems, respectively. The separation ratio of the F_2_ population was close to 9:7 (x^2^ = 0.244, *p* = 0.197), indicating that stem color traits may be regulated by the two major effect genes (Table 1).

### 2.2. Determination of Pigment Content

**Figure 2:**
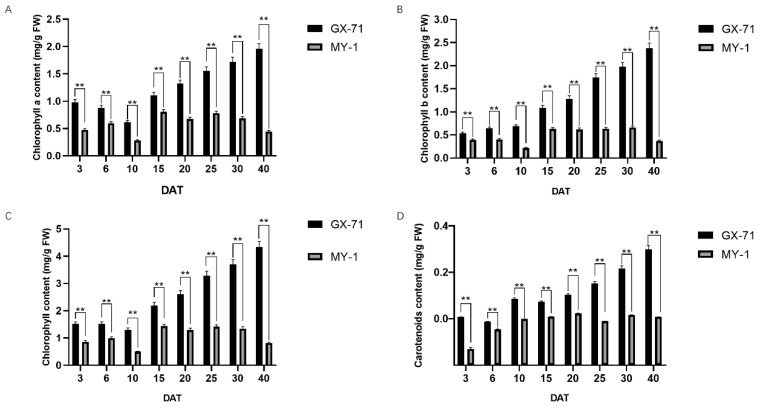
Analysis of pigment content of parental stems at 3–40 d after transplanting (GX-71 and MY-1): (**A**) chlorophyll a, (**B**) chlorophyll b, (**C**) chlorophyll, and (**D**) carotenoid contents. **, *p* < 0.01.

The chlorophyll and carotenoid contents of the parental stems at different developmental stages were measured at 3, 6, 10, 15, 20, 25, 30, and 40 d after transplanting. At 15 d post-transplant, the chlorophyll content of the green-stemmed parent GX-71 gradually increased, whereas that of the white-stemmed parent MY-1 did not change significantly (Figure 2A,B). At 40 d post-transplant, the chlorophyll content of GX-71 was more than five times that of MY-1 and the carotenoid content of GX-71 was significantly higher than that of MY-1 (Figure 2C). 

### 2.3. QTL Mapping and Candidate Gene Identification

**Figure 3:**
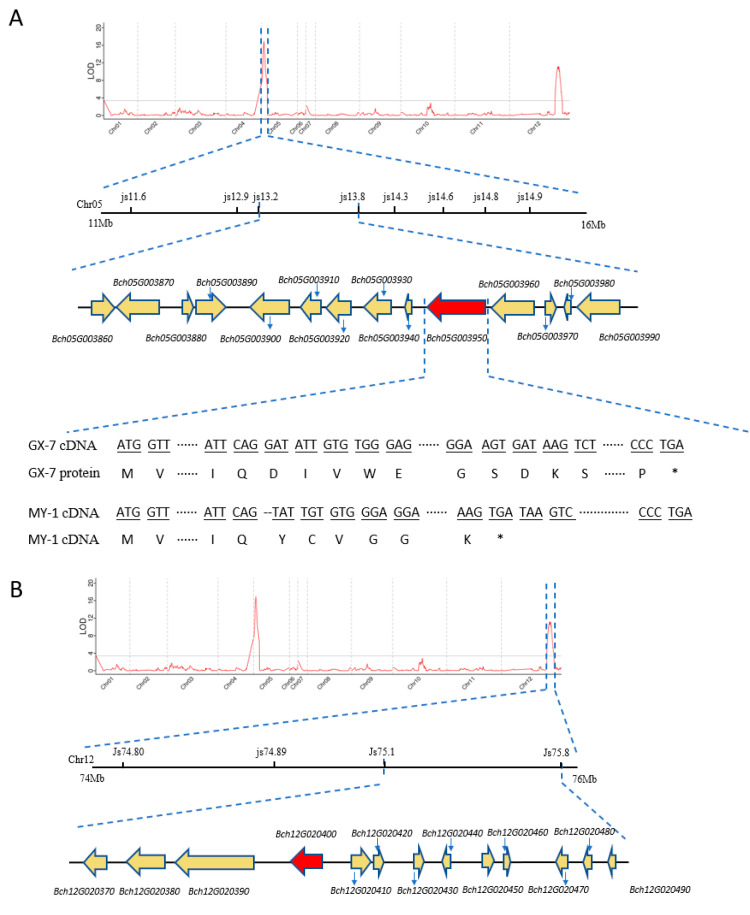
Fine-mapping of the main regulatory gene *BchAPRR2* and secondary gene, *Bch12G020400*, involved in wax gourd stem color. (**A**) Fine-mapping of candidate genes in *qSC5*, which was narrowed to js13.2 and js13.8, with a total length of 633.057 kb, containing 14 genes. Gene sequence analysis of *BchAPRR2* revealed two base deletions. (**B**) Fine-mapping of candidate genes in *qSC12*; *qSC12* was narrowed down to a total length interval of 569.389 kb containing 13 genes.“*” denotes a stop codon.

QTL mapping was performed using HDM and phenotypic data. The genetic map contained 128,9176 variation sites and 1,256,985 markers, with a total distance of 1345.1 cM, divided into 956 bins and 12 linkage groups. Based on the observed phenotypic values of the 105 inbred lines and HDM, QTL mapping of stem color-related traits was performed using the composite interval mapping method. Two QTLs, *qSC5* and *qSC12*, were identified as the main stable QTLs on two chromosomes: Chr05 (11,134,567–16,459,268) and Chr12 (74,618,168–75,712,335), respectively. The explainable phenotypic variation rate and maximum limit of detection of *qSC5* were 36.9% and 16.9, respectively, while those of *qSC12* were 20.9% and 11.2, respectively. Based on the combination of QTL results with unpublished data of the wax gourd reference genome (GX-19) with the GX-71 and MY-1 resequencing data, eight pairs of polymorphic InDel markers were developed for genotyping and linkage analysis of *qSC5*. Finally, the interval, which contained 14 genes, was narrowed to js13.2 (13,205,354) and js13.8 (13,838,411), with a total length of 633.057 kb (Figure 3A). Individuals in the F_2_ population in which qSC5 was pure heterozygote and the stem color was opposite to the expression of the qSC5 genotype were selected and self-pollinated to obtain an F_2-1_ population with 1120 individuals. Four pairs of polymorphic InDel markers were developed to narrow qSC12 to a 569.389 kb region containing only 13 genes (Figure 3B). Gene sequence and annotation analyses (Table 2) of the genes in this region showed that Bch05G003950 exhibited two-base deletions in the coding sequence of maternal plants, resulting in the formation of a premature termination codon (Figure 3A). Additionally, the gene annotation of Bch12G020400 indicated that it encodes phospholipase A(1) DAD1, a protein with chloroplast-like characteristics (Table 3).

### 2.4. qRT-PCR Analysis

**Figure 4:**
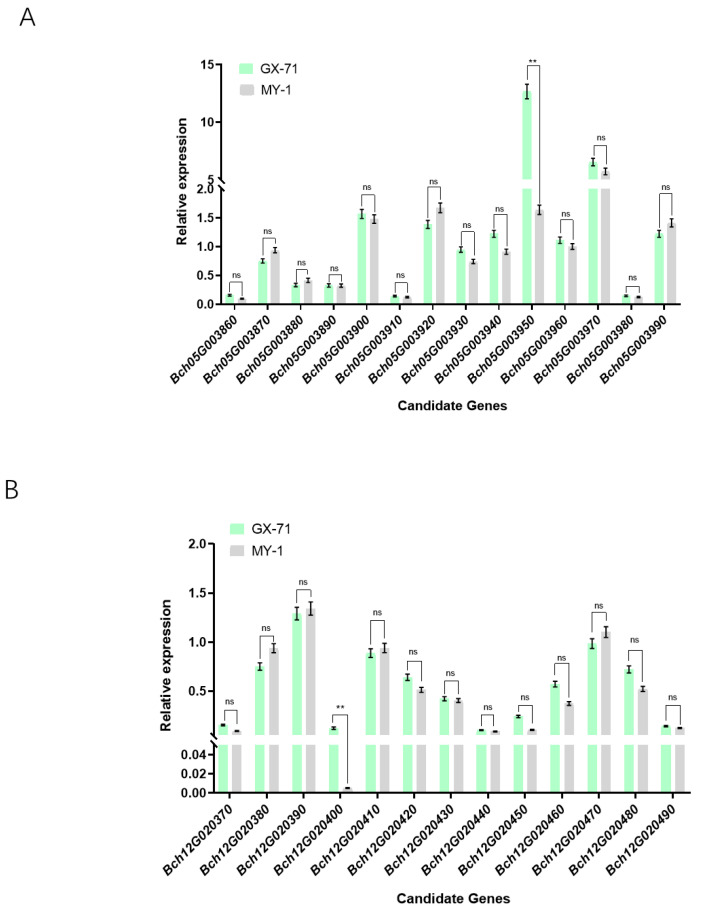
Real-time quantitative expression of candidate genes. (**A**) Expression analysis of candidate genes (**A**) within the reduced *qSC5* interval and (**B**) within the reduced *qSC12* interval in stems. **, *p* < 0.01. “ns”, no significant difference.

To further analyze the candidate genes, qRT-PCR was performed to examine the expression of genes within the parental region. Stem segments with stable stem color traits were selected from the transplanted 40DAT as materials for differential expression analysis of all genes in the region. Differential expression analysis showed that only the expression of *Bch05G003950* and *Bch12G020400* differed significantly compared with those of their parent plants. First, *Bch05G003950* exhibited increased relative expression in the parental plant GX-71 and reduced relative expression in the maternal plant MY-1 (Figure 4A). Second, *Bch12G020400* expression was negligible in the maternal plant but that in the parental plant exhibited significant variation (Figure 4B). These results indicate that *Bch05G003950* and *Bch12G020400* may be key genes regulating the stem color in wax gourds.

### 2.5. InDel Marker-Assisted Breeding

**Figure 5:**
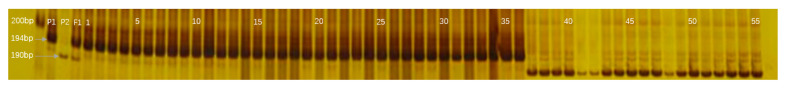
InDel marker used to verify the 55 parts of the wax gourd germplasm resources. P1 and P2 represent GX-71 and MY-1, respectively; 1–36 represent green-stemmed wax gourds and 37–55 represent white-stemmed wax gourds.

An InDel marker tightly linked to *BchAPRR2* was developed to verify the consistency of the stem color genotypes and phenotypes of the experimental wax gourds (Supplementary Table S1). In total, 55 wax gourd germplasm resources with extreme stem color traits were selected to verify the InDel marker. Among them, 34 and 21 had green and white stems, respectively. The results showed that 36 and 19 germplasm resources were consistent with green-stem male parent GX-71 bands and white-stem female parent bands, respectively (Figure 5). The coincidence rate of the genotype and phenotype was 81.8% (Supplementary Table S2).

## 3. Discussion

The stem color of wax gourds is of great significance for their development and fruiting. Differences in chlorophyll content in the stems of wax gourds lead to different stem colors. Numerous studies have been carried out on pigments but the stem color in Cucurbitaceae has been sparsely investigated. Studies have shown that pigment type and content can determine the color of plant tissues [23] and that main pigment types can differ by tissue. In eggplant peel, chlorophyll and anthocyanins are the primary pigments that determine color [24]. In Chinese kale, which has purple stems, anthocyanin is the main pigment that governs stem color. *BoDRF*, which controls the purple stem color of Chinese kale, is located at a 0.32 cM interval. However, the insertion of a base in this gene leads to a frame shift mutation, resulting in the production of green stems [25]. Chlorophyll is the main component of green tissues; therefore, a lack of chlorophyll in green tissues leads to plant albinism [26].

In this study, the main pigment controlling the stem color of wax gourds was determined to be chlorophyll; therefore, we observed and measured chlorophyll changes in wax gourd plants for 40 d post-transplantation. During this period, the stem chlorophyll contents of parental GX-71 and maternal MY-1 plants significantly differed.

### Molecular Mechanism Regulating the Stem Color of Wax Gourds

Various family factors, including the GLKs, MYB, APRR, and other families, are reportedly involved in the regulation of pigment traits. For example, in strawberries, FaMYB controls the formation of red strawberries by regulating flavonoid biosynthesis during the late stage of fruit development. Wang et al. [27] inserted eight bases in a specific variant allele of *FaMYB10*, which resulted in the formation of a premature termination codon and the production of white octoploid strawberries. Additionally, the GLK family transcription factors, which are key nodes in the plant regulatory network, trigger the expression of photosynthesis-related nuclear genes [28]. For example, the chlorophyll regulatory factor BGP4 reportedly affects chlorophyll contents by influencing the conduction of light signals, the interaction with the GLK transcription factor, and the inhibition of transcription factor activity [29]. In this study, the main regulatory gene for wax gourd stem color was *BchAPRR2*, which belongs to the APRR family. In squash, the deletion of the chromosomal fragment of the repeated locus on APRR2 leads to the synthesis of chlorophyll in stems, which affects the production of white-stem varieties of squash [7]. Moreover, APRR1 is related to pigments [30] and Nong et al. [31] reported that APRR2 is closely related to the skin color of wax gourds. Furthermore, allele variation in *BchAPRR2* across varieties of wax gourds affects the plant chlorophyll content and structure. Moreover, APRR family genes are reportedly involved in the regulation of circadian rhythms in *Arabidopsis* [32]. *APRR9* of the APRR family encodes regulatory factors related to light-induced photoresponses and participates in the regulation of the circadian rhythm in *Arabidopsis* [33]. In the present study, the annotation of *Bch12G020400* revealed that it encodes phospholipase A(1) DAD1, a chloroplast-like protein. In *Arabidopsis*, the DAD1 protein is chloroplast phospholipase A1, which is a fluorescent green fusion protein mainly located in chloroplasts [34]. Therefore, a high expression of *Bch12G020400* may promote the production of the green fluorescent protein DAD1, thereby affecting the stem color of wax gourds.

## 4. Materials and Methods

### 4.1. Plant Material and Phenotypic Evaluation

In this study, 105 recombinant inbred gourd lines were constructed. Green-stemmed GX-71 (male parent) and white-stemmed MY-1 (female parent) plants were utilized as parents to produce populations for QTL localization. The F_1_ generation was hybridized and self-pollinated to obtain the F_2_ generation, comprising 2218 plants. Individuals with different stem colors from F_2_ without *qSC5* exchange were selected for self-pollination to obtain the F_2-1_ line. F_2_ was used for fine-mapping genes in *qSC5* and F_2-1_ was used for fine-mapping genes in *qSC12*. Simultaneously, 55 germplasm resources were used to verify the InDel markers developed in this study. All materials were procured from Nanning Kenong Seed Industry Co., Ltd., China, and planted at the Nanning Yong’an Wax Gourd Experimental Base (E 108°51′, N 22°48′) from July 2022 to July 2023 for growth under natural light. The parents of the hybrid combination and F_2_ generation plants were labeled with serial numbers. The wax gourd plants used for phenotypic determination were collected 40 d after transplantation, which ensured that chlorophyll was in a stable state. The stem color of wax gourds was visually evaluated in the field and categorized as white and green. Specifically, a stem color resembling the white stem of the mother plant was recorded as white and a color resembling the green stem of the father plant was recorded as green.

### 4.2. Extraction and Determination of Chlorophyll and Carotenoid Contents

Stems were collected at the same plant height at 0, 3, 6, 10, 15, 20, 25, 30, and 40 d after transplanting. The stems were cut into ~3 cm-long segments using a knife and ground into a powder with liquid nitrogen. Powder (1.0 g) was placed into a 15 mL centrifuge tube and shaken in a light-proof environment at 200 rpm for 12 h to extract pigments. The chlorophyll a, chlorophyll b, and carotenoid contents were evaluated at 665, 649, and 470 nm, respectively. The respective equation was derived from Li et al. [35] (Supplementary File S1). 

### 4.3. DNA Extraction

A plant genomic DNA extraction kit (Solarbio Science, Beijing, China) was used to extract genomic DNA from the young leaves. The obtained DNA was quantified using an ultra-micro spectrophotometer (K5800, KAIAO, Beijing, China) and its integrity was evaluated using 1.2% agarose gel electrophoresis.

### 4.4. QTL Mapping and InDel Marker Analysis

QTL mapping was performed using HDM and phenotypic data. The map contained 1,289,176 variation sites and 1,256,985 markers and had a total distance of 1345.1 cM. QTL mapping was performed using the composite interval mapping method and QTL Cartographer (version 1.17j) software (https://brcwebportal.cos.ncsu.edu/qtlcart/ accessed on 16 February 2023) for preliminary localization. InDel markers were developed for genotyping and linkage analysis using Premier 5.0 software based on the whole-genome resequencing data of the parents and 105 inbred lines. Several developed InDel polymorphic molecular markers were used in combination with the F_2_ population data for fine-mapping genes in *qSC5*. Other developed InDel polymorphic molecular markers were used in combination with F_2-1_ data for fine-mapping genes in *qSC12*.

### 4.5. RNA Extraction and Candidate Gene Prediction Analysis

Total RNA was extracted from GX-7 and MY-1 using the EastepSuper Total RNA Extraction Kit (Promega, Beijing, China) according to the manufacturer’s instructions. Gene sequence comparisons were performed between the candidate gene sequences, which were obtained via the resequencing of the parents and 105 inbred lines. The corresponding gene annotations were searched for in the existing wax gourd reference genome, GX-19. Gene sequence alignments were produced using DNAMAN v.9 software (Lynnon Biosoft, San Ramon, CA, USA). Finally, gene sequence analyses combined with gene annotation analysis were used to screen candidate genes involved in the regulation of wax gourd stem color.

### 4.6. qRT-PCR Analysis of Candidate Genes

qRT-PCR was used to quantify the differential expression of candidate genes in the parent plants. First, total RNA was extracted from parental stem segments 40 d after transplantation and reverse-transcribed using a reverse transcriptase RT Master Mix (Takara, Beijing, China). Primer sequences to amplify the reference genes *CAC* (*Bch05G003650*), *Bch05G003950,* and *Bch12G020400* and candidate genes were designed using Premier 5.0 (Supplementary Table S1). The qPCR analysis was performed using a premixed SYBR Green quantitative PCR reagent and an Applied Biosystems 7500 qRT-PCR system (Foster City, CA, USA). Each experiment was repeated three times and relative expressions were determined using the 2^-∆∆Ct^ method [36]. Differences in relative expression were analyzed using GraphPad Prism 9 (GraphPad Software, San Diego, CA, USA).

### 4.7. Molecular Marker-Assisted Breeding

A pair of InDel markers tightly linked to the predicted main regulatory gene *BchAPRR2* was designed using Premier 5.0. The primer sequences are listed in Supplementary Table S1. Combined with developed markers, 55 wax gourd germplasms from the parent plants and F_1_ with extreme stem color traits, including 34 green stems and 21 white stems, were used for molecular marker-assisted and stem color accuracy verification experiments (Supplementary Table S2).

## 5. Conclusions

Two-base deletions of the main regulatory gene, *APRR2*, of stem color led to a frame shift mutation, which may change the structure and function of the protein. The high expression of the secondary regulatory gene *Bch12G020400* promotes the production of the green fluorescent protein DAD1 in the chloroplast. *BchAPRR2* and *Bch12G020400* may affect the synthesis, structure, or function of proteins in chloroplasts via complex molecular regulatory mechanisms, ultimately leading to stem color differences in wax gourds. *BchAPRR2* and *Bch12G020400* were found to be related to the stem color trait of wax gourd. However, further research on the molecular mechanisms of *BchAPRR2* and *Bch12G020400* regulating wax gourd stem color may face some difficulties, mainly due to the incomplete establishment of the wax gourd genetic transformation system. Our study provides a theoretical basis for further research on these mechanisms and a reference for stem color regulatory genes of other gourd species. Our findings will help genetically improve wax gourd stem color and specific germplasm resources. 

## Figures and Tables

**Table 1:** Distribution of stem color in wax gourd populations.

Plant Type	Total No.	Green (Light Green)	White	X^2^ (9:7)	*p*
GX-71	10	10	-	-	-
MY-1	10	-	10	-	-
F**_1_** population	10	10	-	-	-
F**_2_** population	786	449	337	0.244	0.19

**Table 2:** Genes within the narrowed *qSC5* interval.

Gene ID	Nonsynonymous Mutations in Coding Sequences	Physical Location	Gene Annotation
*Bch05G003860*	Yes	Chr5:13207272–13212788 (+)	protein NRT1/ PTR FAMILY 7.3-like
*Bch05G003870*	Yes	Chr5:13213544–13235133 (−)	Pyruvate kinase isozyme G
*Bch05G003880*	Yes	Chr5:13274698–13276057 (+)	-
*Bch05G003890*	No	Chr5:13276096–13281871 (+)	Protein ABCI7, chloroplastic
*Bch05G003900*	Yes	Chr5:13341180–13346696 (−)	Probable ribose-phosphate Pyrophosphokinase 1-like
*Bch05G003910*	Yes	Chr5:13367114–13372139 (−)	Probable tobamovirus multiplication protein 2B isoform X1
*Bch05G003920*	Yes	Chr5:13438283–13441573 (−)	Probable peroxidase 20 isoform X1
*Bch05G003930*	No	Chr5:13441626–13447889 (−)	Tobamovirus multiplication protein 2A-like
*Bch05G003940*	Yes	Chr5:13484454–13484678 (−)	-
*Bch05G003950*	Yes	Chr5:13491669–13499644 (−)	Two-component response Regulator-like protein APRR2
*Bch05G003960*	Yes	Chr5:13676056–13689823 (−)	MSC domain-containing protein
*Bch05G003970*	No	Chr5:13805666–13806021 (+)	Vacuolar protein sorting-associated protein 55 homolog
*Bch05G003980*	No	Chr5:13810161–13810902 (−)	Uncharacterized protein LOC111455443
*Bch05G003990*	Yes	Chr5:13825779–13838208 (−)	Transcription factor MAMYB

**Table 3:** Genes within the narrowed *qSC12* interval.

Gene ID	Nonsynonymous Mutations in Coding Sequences	Physical Location	Gene Annotation
*Bch12G020370*	Yes	Chr12:75155250–75160607 (−)	Probable H/ACA ribonucleoprotein complex subunit 1-like
*Bch12G020380*	No	Chr12:75215164–75221452 (−)	RGG repeats nuclear RNA binding protein A-like isoform X2
*Bch12G020390*	No	Chr12:75225625–75297961 (−)	Probable glycine—tRNA ligase, chloroplastic/mitochondrial 2 isoform X1
*Bch12G020400*	No	Chr12:75332840–75335150 (−)	Phospholipase A(1) DAD1, chloroplast-like
*Bch12G020410*	No	Chr12:75409841–75418358 (+)	Carotenoid cleavage dioxygenase 7, chloroplastic
*Bch12G020420*	No	Chr12:75418509–75419660 (+)	Probable major pollen allergen Ole e 6-like
*Bch12G020430*	No	Chr12:75498816–75499589 (+)	Probable major pollen allergen Ole e 6-like
*Bch12G020440*	No	Chr12:75510818–75511148 (−)	-
*Bch12G020450*	No	Chr12:75522841–75523897 (+)	Probable major pollen allergen Ole e 6-like
*Bch12G020460*	No	Chr12:75529773–75530669 (+)	Probable major pollen allergen Ole e 6-like
*Bch12G020470*	No	Chr12:75548538–75551483 (−)	-
*Bch12G020480*	No	Chr12:75557730–75559794 (−)	Pentatricopeptide repeat-containing protein At4g01570
*Bch12G020490*	No	Chr12:75564027–75565069 (−)	Probable uncharacterized protein LOC103498828

## Data Availability

The datasets presented in this study can be found in online repositories. The names of the repository/repositories and accession number(s) can be found below: CNSA accession number: CNP0004715, https://db.cngb.org/search/?q=CNP0004715 accessed on 5 October 2023.

## Supplementary Materials

The following supporting information can be downloaded at: https://www.mdpi.com/article/10.3390/plants13131804/s1, Table S1. Primer List, Table S2. Molecular marker validation of 55 germplasm resources, Supplementary File S1. Candidate gene sequences.
