# Supplementary material for: Quantitative Trait Loci Sequencing and Genetic Mapping Reveal Two Main Regulatory Genes for Stem Color in Wax Gourds
Source: Plants (Basel). 2024 Jun 29;13(13):1804. doi: 10.3390/plants13131804 (PMC11244448; doi:10.3390/plants13131804)
Supplement: Supplementary file 1 [file plants-13-01804-s001.zip › Supplementary File S1.pdf]

Pigment calculation equation

Ca (mg/L) = 13.95 × A665 − 6.88 × A649, (1)
Cb (mg/L) = 24.96 × A649 − 7.32 × A665, (2)
Cx.c (mg/L) = (1000 × A470 − 2.05 × Ca − 114.8 × Cb)/245, (3)
Pigment content (μg/g) = [1000 × pigment concentration (mg/L) × extracted liquid product (L) × dilution factor]/sample weight (g), (4)

Total chlorophyll content (μg/g) = Ca content + Cb content, (5)

Ca is the concentration of chlorophyll a, Cb is the concentration of chlorophyll b, and Cx.c is the carotenoid concentration. A665 is the absorbance of chlorophyll a at 665 nm, A649 is the absorbance of chlorophyll b at 649 nm, and A470 is the absorbance of carotenoid at 470 nm.

Linked marker for qSC12

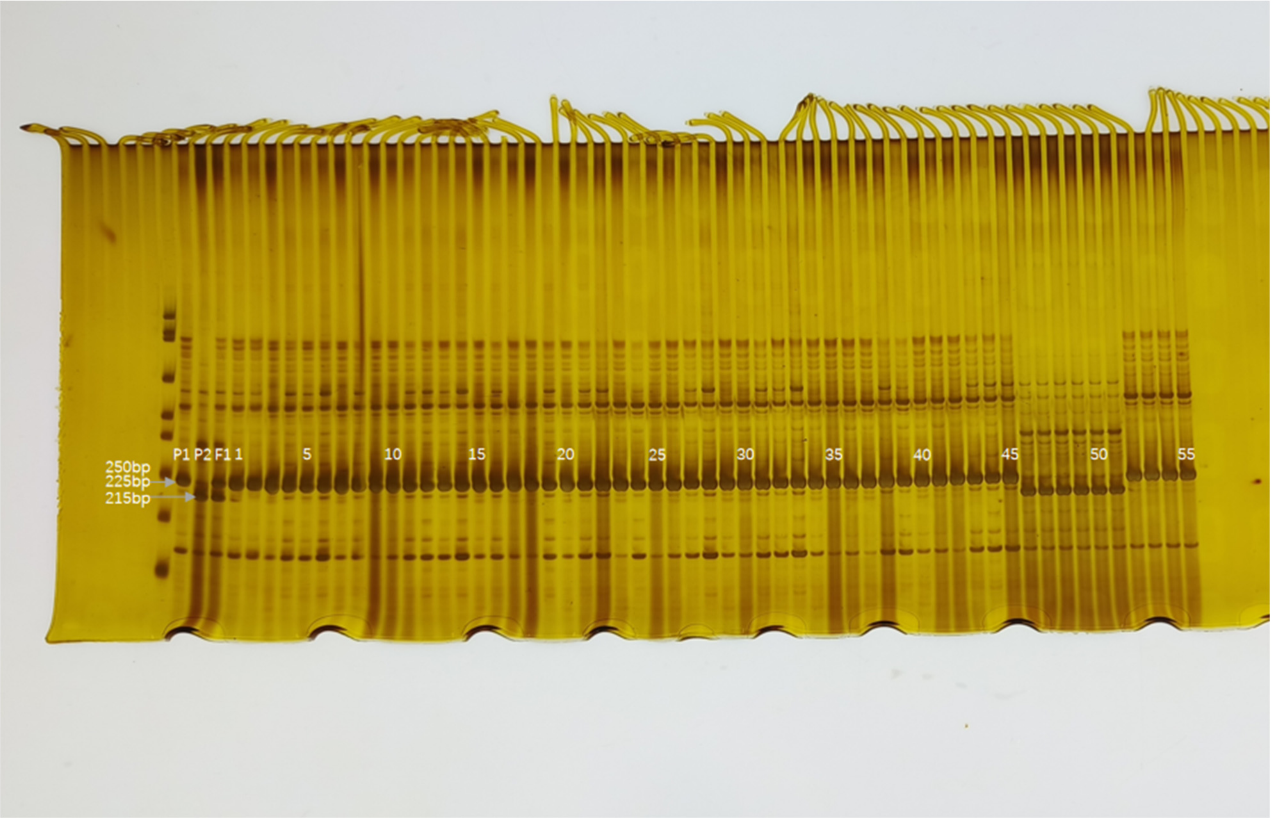

The coincidence rate of the genotype and phenotype was 58.1%. (Supplementary Table S2).

Sequence data of all gene

qSC5

Bch05G003860

ATGCCCTCAAGACGAAAATCCAGAAAACTCATATTACAAGAGGAAGAGAGGGAAATTTGTACTCTTGATG
GGACTATTGATTGGCATGGCCAGCCAGCAATCAGATCAAAATCTGGAGGATGGGTTGCGGGAATCATCAT
ACTCTTGAATCAAGGTCTTGCAACTCTTGCGTTCTTTGGGGTTGGTGTGAACTTGGTGCTGTTCCTGACA
AGGGTTTTGCAGCAAAACAATGCCGATGCGGCCAACAGTGTGAGCAAATGGACCGGGACAGTGTACATCT
TCTCTCTTGTTGGGGCTTTCCTCAGTGATTCATACTGGGGAAGGTACAAAACTTGTGCCATTTTTCAGAT
CATCTTTGTCATTGGCCTGGTTTCACTTTCCATAACATCACAGCTCTTCCTGATCAAACCAAAAGGTTGT
GGGGATGAGGATACTCCATGTGAGTCACATTCAAGATTGGGGAATGCATTGTTTTACCTCTCCATTTACC
TTATTGCACTGGGAAACGGGGGATATCAACCAAATATCGCCACATTTGGAGCAGACCAATTTGATGAAGA
GGACTCCACAGAGGGGCACTCAAAAGTGGCATTCTTTAGCTACTTTTACTTGGCGCTTAACTTGGGTTCT
CTCTTCTCAAACACCATTTTAGGGTACTTTGAAGATGTAGGAGTGTGGGCTTTGGGCTTCTGGGTGTCAA
CTGGCTCAGCTTTTGCAGCATTGTTGTTGTTTCTTTGTGGAACCCCAAGGTACAGGCACTTCAAGCCAAG
TGGTAACCCTCTCTCAAGATTCTGCCAAGTCATTGTTGCTGCAGCTAAGAAATCAACGGTCAAGATGCCA
CCGAGTGAAGATGACCTATACAGTGTTGATGCTAAGGAGGACTGTTCAATGAATCGCGGTAGGAAGATAC
TTCACACCCATGGATTCAAGTTTCTGGATAGGGCAGCATATATCTCATCAAGAGATTTAGATGACCAAGG
CCGAGGAATTGACGATCCTTGGCGCCTCTGTCCGATCACACAAGTGGAAGAGGTCAAGTGCATTTTAAGA
TTACTTCCTATTTGGCTCTGCACCATAATCTACTCTGTGGTCTTCACTCAAATGGCTTCCCTCTTTGTCG
AGCAAGGCGCTGCCATGAAAACTATTGTTTCAAACTTCCATATCCCACCTGCAAGCATGTCCAGCTTTGA
CATTCTCAGTGTGGCATTTTTCATTTTTCTTTACCGACGAGTCCTAGAACCACTTGCAGGAAAACTCAGA
AAGTCGGATTCTAAAGGGCTTACTGAGCTCCAGAGAATGGGAATCGGTCTCATCATAGCAGTGATGGCAA
TGGTTTCAGCAGGAATTGTAGAGTGTTATAGGCTAAAGTACGCCAAAGGTGACTGCACACACTGTGAAGG
TTCAAGCTCCTTGAGCATCTTTTGGCAGGTTCCACAGTATGCATTTATAGGAGCTTCCGAGGTTTTCATG
TACGTGGGACAATTGGAGTTTTTCAATGCACAAACCCCAGATGGATTGAAAAGCTTTGGTAGTGCACTTT
GTATGACATCAATCTCTTTGGGAAATTATGTCAGTAGCCTTCTGGTCACTATGGTTATGAAAATATCAAC
AGAGGACCACATGCCAGGATGGATTCCAGGAAACCTGAATAGAGGTCATCTAGATAGATTTTACTTCCTC
CTAGCTGGATTGACTGCAATCGACTTTGCAGTGTATATTATGTGTGCTAAATGGTACAAATGTATCAAAT
TGGAGGGCAAGTGTCAAGAAAATGAAGACCCGGGGCACATCAAGGTCTGA

Bch05G003870

ATGGCGACGTTTAATCTCTCTACTGGAGTGCCTCTGCTGAAATATGATTCTACCAGAGTCGCCGACCGCC
TTGCGTCGTGTAGAATCGTCTCTGATGCTTTTGGTCTTGAAGCGAAGAGTGGAAGGATGTGTTTGCAATC
GAAGCAAATCGCTTCGGTTAGATGCTTGAGAATAGCCGAGCAGCGGGAAATTGCGTCGTATAATGGCTCT
TTGGATACTGATCAGGACGTTTCAAACTCTACCTTTGAGCTTCAGTCTAATGCATTCCCTCTTAGCAGGA
CGAAGTTAACTACAAAATCTCGAAGGAAAACTAAGATAGTCTGCACCATTGGCCCTTCGACAAATTCACG
GGAAATGATATGGAAATTGGCAGAGACTGGGATGAATGTGGCACGTTTAAACATGTCCCATGGGGATCAT
TCTTCCCACCAGAAAACCATTGATTTGGTTAAGGAATATAATGCCCAATTTACGGACAAAGTTATAGCCA
TTATGCTTGACACGAAGGGCCCTGAGGTTCGAAGTGGAGATGTACCTAAACCAATCTTGCTCAAAGAAGG
ACAAGAATTTAACTTCACAATCAAAAGAGGAGTCAGCACAGAAGACACTGTTAGTGTCAACTATGACGAC
TTTGTAAATGATGTAGAAGTTGGAGATACTTTACTTGTTGACGGTGGAATGATGTCATTGACTGTTCAGT
CGAAGACAGATGATTTGGTTAAGTGTGTAGTCATTGATGGCGGTGAACTCAAATCGAGGCGTCATTTGAA
TGTTCGTGGAAAAAGTGCGACATTGCCTTCGATAACAGAAAAGGACTGGGAAGATATAAAGTTTGGGGTG

GATAATCAGGTTGATTTTTATGCTGTTTCTTTTGTGAAGGATGCTAGAGTGGTTCATGAGCTGAAAGACT
ATCTGAAAAGCTGTAATGCAGATATTCATGTGATTGTAAAAATTGAAAGTGCAGACTCAATACCTAATCT
TCATTCGATACTTTCAGCATCAGATGGGGCAATGGTCGCACGTGGAGACCTTGGGGCTGAACTTCCAATT
GAGGAAGTTCCTTTATTGCAGGAGGATATTATCAAAAGATGTCGAACCATGCAGAAACCAGTTATTGTGG
CAACAAACATGCTGGAAAGCATGATTGATCATCCCACGCCAACTAGAGCCGAGGTTTCTGATATTGCTAT
TGCAGTACGTGAAGGTGCTGATGCAGTCATGCTTTCTGGAGAAACTGCTCATGGAAAGTATCCATTGAAG
GCTGTGAAAGTGATGCATACTGTGGCTTTGAGGACGGAATCTAGTCTGCCAATTAGTCCTACTACTCCAA
ATCCTTTGAGTGTCCACAAGAGCCATATGGGAGACATGTTTGCTTTCCACGCCACCACTATGGCCAACAC
CCTTAGTACTCCTATCATTGTTTTCACAAGAAGTGGCTCCATGGCAATACTCTTAAGTCATTATAGGCCC
TGCTCTACTATCTTTGCCTTCACTGATGACGAAAGAATTAAACAAAGGCTGGTGCTTTATCATGGGGTCA
TGCCCATCTACATGCAATTTTCAAATGATGCAGAAGAGACGTTCTCCAGAGCACTCGAACTATTGCTGGA
TAAGGGTCACGTGGCAGAGGGAGACCACGTTACGCTTGTTCAAAGTGGAGCCCAACCAATTTGGCGGAAA
GAATCTACTCACCACATTCAAGTACGTAGGATCCAAGGTTGA

Bch05G003880

ATGGCCAAACCGAAACTGAACCGCTTTTGTTTATACATGAAATCAAACCGGTTCAATTTACCGCACATCA
AAAGAAGAAAGGCAGCCCAGCCACACCAATCTCGCCGTCTTTCTTCTCTCTTCATTTCACTGTTTCCGTT
GCCGGCCGTCGGGAAAGAAAATGGCGACCTGTTTTTGACGAAAAATGGTGAAGATCCAGCTAGTTGTGTT
GCGGCTAAAATGAAACTCTTGATCATGAGTATCAAATCGAGACAGTGA

Bch05G003890

ATGGCTACCTTCACATTTACTCCCCATATTGCTAGGCTTCCAACGCTATCCTCTTCTTCAAAACGGAAAC
CCAAATTCAATTCCATTAAAACCACCAAAGTCTCCCTTCAAACTTCCCCACCTTCACTCTCTGATCCCTT
TGTTCTTCAACTCGCCGAGACTCTCGAAGACTCGCTCCCTTCTTCTTCTTCTTCTTCTTCTTCATTGCCT
TTTCCTCTTCAGAAACTCAGGGAATCTTCCGCTGAGACTCTCCTTTCGACTCCGTGGCCCTCTCGCAGAG
ACGAGCCGTTTCGTTTCACAGATGTTTCTTTCATAAAACAGTCCCAAATCCACCCAATCTCGAATCCGCC
ACAGTCTTCCCAATTATCTAACATTCCTGTAGAAACCCAGTTCGCCAATGTTGTAATTGTTGATGGTCAT
TTCGTCAATTCAGTTTCCAATCTGACAGAATTGCCAAATGGGGTTTATGTGGGTAGCTTCATTGACCTCC
CTTCCGAGAGTATTGCGAAGAGGGTGTCTGAGTTTGTTGATGGGAAGTTCGTGGGGGACTTGTTTTGGTC
CATTAATGGTGTTGGAGCTCCAGATTTGACTGTGGTCTATGTACCTGCTGGGTGTAAGGTAGAGAATCCA
ATCCATTTTAGGTATTTTTCTATCAATGGGGGCGAGGAAGGGTCAAAGGAATTGGCTGTTTCGAATCCTA
GAGTGTTGGTTTTGGTGGAAAATGGAGGGGAGATTGAAATTATTGAGGAGTTTTTGACTGGAGATGGGGG
TAGGTCTTATTGGGCAAATCCTGTTTTGGAGGTGGTAATTGGAAGTGGAGGAAAGGTTAAACATTCTTAT
ATTCAGAATCAGTCTTTAAATGCTGCACATATCAAATGGACTTCTGTTCAGCAGGAATCAACCAGTGCCT
ATGAGCTTGTGGAGATTAGTACGGGTGGAAAACTAAGTAGACACAATGTCCATATCCAACAGTTGGGACC
AGAGACTACGACAGAATTATCAACACTGCACTTATCAGTTGGCAATCAAACACAAGATCTACACAGTAGC
CTAATGCTCGACCATCCACGAGGATATTCTCGACAGCTTCATAAATGTATTGTAGCCAATCCCCAAGGAC
AAGCCGTATTCGATGGTAACATAAAAGTCAACAGATATGCACAACAAACGGATGCAGGACAACTAACAAG
GAGTCTTCTTCTAGAACCTCGAGCAACCGTGAATGTGAAACCTAATCTTCAAATCATTGCTGACGACGTC
AAGTGTTCACACGGAGCTGCTATAAGCGACTTGGAAGAAACCCAACTCTTCTATTTCCAGGCTCGTGGAA
TAGACTTGGAGACAGCAAGGAAGGCTCTAATATTTTCTTTTGGAGCCGAGGTGATCGAACGGCTACCTTC
CCCTTCGGTCCGGAAGAGAGTTGAGAATCATATAAAAGAATTGTTGAACCCCACACTAGAAAGATCCTAA

Bch05G003900

ATGGCTTCTATGGCTGCGTTGACTCTTCCGTCTTCTTCTTCGTCGTCGTCGTTGTCGTCTCTGTTCTCTG
TTGGCTCCCTCGACCATGGTGCTTTTGTTTCGAACGAGTCTCGCATTCGAATGTGTTCCAATGGCGGAGT
TAGATGTGATCTGTCCGAGTCTTTGAAGCATGTGAACGGTAAACCAACAATTCCAATTGTTACTGAACGG

ACGCTACCCAAGTTCTTGGAATCAGCACGCATGGAGCAAAGAGTTGATAGAAGTAGTACGCGTTTGAAAT
TGTTTTCTGGCTCTGCAAATCGTTTACTTTCACAGGAAATTGCCTGCTACATGGGGCTGGAGCTTGGAAA
GATTAACATTAAGCGGTTTGCTGATGGTGAAATATATGTTCAATTGCAAGAAAGTGTTAGGGGATGTGAT
GTATTCCTAGTACAGCCAACCTGCCCCCCAGCTAATGAGAATCTCATGGAGCTTTTAATTATGATAGATG
CTTGTAGGAGAGCCTCGGCCAAAAATATTACTGCTGTGATTCCATATTTTGGATATGCTAGAGCCGATAG
AAAGACTCAAGGGCGTGAATCGATTGCTGCGAAGCTTGTTGCCAACATCATTACTGAAGCTGGGGCAAAT
CGTGTTCTTGCTTGCGATCTTCATTCTGGACAATCCATGGGTTACTTTGATATTCCTGTGGATCATGTAA
ATTGCCATCCCGTGATTCTTGACTATCTTGCCAGCAAGAGGATTTGTTCTAGTGATCTGGTAGTGGTTTC
TCCTGATGTTGGAGGAGTTGCCAGAGCACGTGCTTTTGCTAAAAAATTATCTGATGCACCATTAGCCATA
GTGGATAAGAGGCGCCATGGTCACAATGTTGCTGAGGTAATGAACCTGATTGGTGATGTAAAAGGAAAGG
TTGCAGTTATGGTTGATGACATGATTGACACAGCTGGAACTATTACAAAAGGGGCTGAACTGCTACATCA
AGAGGGGGCCAGAGAAGTTTATGCTTGTTGCACCCATGCTGTTTTCAGCCCCCCTGCCATTGAGAGGTTG
TCCAGTGGCTTTTTTCAAGAGGTGATTGTTACAAACACAATTCCAGCCCCCAAAAGTCACTTCCCTCAGT
TGACAGTCCTCTCAGTCGCAAACTTGATGGGTGAAACCATTTGGCGTGTTCATGATGATTGTTCGGTGAG
TAGTATTTTTCAATGA

Bch05G003910

ATGGCGACAGCTTCCGCATCAGGTCGAAGCAATAGAGATGGAACAGCCAAAGCCATGGTGGCCGACCACA
TTTCTCAGTCGGTACGATCCACTTCAAATCTCCTTCATCTCATGCAGCAATCTTCTTCGGCTCAGGCTCA
GTTAACAAAGTTGCCAAAGAATCTTTTGGCGAAAACACCTACTATTAAAAATATTGGGAAAATCTTAGAG
CAGATGCCACAGGTGGTTTCGTCATTAGATGCATATGTTGAGAAGGGACTAGAAAGCATTCCTCATTTGC
AGACTGTCGTGCAATTACTTACAAACATGGAAAGCTCCCAGCTGAAATCTCTGTCTCAATTCCAACAACC
TCATGAGGAAAGCGAATCTCTTCGTCAACTGAAAGATGTGGACTAA

Bch05G003920

ATGGAGAGTTTGAAGATATTAGTGGTTGTAATATTTGGTGTGGTTATGCATGGAATTGGAAGTTTAGGTG
GACTTTTGGTTTTTGACTATTACAAAGAAACATGCCCTTTTGTTGAAGATATTGTTCGACGACAAGTCGA
GATTGCTGTGCTTAAAGATCCTCGAATGGCTGCTTCACTCCTTCGCTTGCATTTTCATGATTGTTTTGTT
ATGGGATGTGATGCATCCGTTCTTCTAGATAGCAATGATGAGATGGTGAGCGAAAAGCAAGCTGCTCCAA
ATTTAAACTCTCTTCGTGGTTTTAGTGTTATCGATGAGATAAAATACATATTGGAAGAAGCTTGTCCTTA
TATCGTGTCTTGTGCTGATATTTTGACCATTGTTGCTCGTGATGCTGTTGTACTCAGAGGGGGACCTGAA
TGGCCAGTGTTGTTGGGAAGGAAAGACTCCTTGAAAGCAAGCTTTGATGGAGCCAACAAATACATTCCTT
CTCCCAATTCCTCCCTTGAGACTCTAATTGCCAATTTCCAACAACAAGGCCTTAACGTTGGAGACTTGGT
TGCTCTATCAGGAAGCCACACCATAGGGAAAGCAAGATGCTTGAGCTTTAGACAAAGAGTATACCAAATG
AATCAAGAAGAAGAATATGATAGATACAAGAGATACAACATATACAGAAGAATTTTAAGGTCAATCTGCC
CTAAAACAGGGCAAGATCAAAGGGTAGCACCCTTAGATTTTAGAACACCTGCAAGATTTGACAACCATTA
TTTCCTCAACATTCTTGAAGGCAAAGGGCTTTTGGGGTCTGACAATGTGCTCATAACCCAAGACTATGAA
GGTGAAATTACAAGACAAGTTTGGAGCTATGCCTCTGACCAAACCCTTTTCTTTGATTCCTTTGTTAAGT
CCATTATCAAGATGGGGAATATCAATGTCCTCACTTCCTATCAAGGTGAAGTCAGAAGGAATTGTAGGTC
TACCAATCACTAG

Bch05G003930

ATGGCCTGCAGAGGGTGCTTTGAGTGCCTATTGAAGCTTCTGAACTTCTTCTTATCCGTGCTGGGTCTTG
CCGTGGTGGGCTATGGGATTTACTTGTTGGTTGAGTACTTGCAATCTCCTAGTGATGTTCCAGGACCTTC
GTTGAGTGGTGATCATGATCTGGTCCAGCTTGGTCGACCAATGCTAATGGCAGTGTCTCTGTCTTCTAAC
TTCTTTGATAACCTTCCAAAAGCCTGGTTCATATACTGGTTCATTGCTGTGGGAGTCATTATCTTTGTTG
TGTCTTGTTTTGGGTGTATTGGAGCTGCAACGCGTAATGGATGCTGTTTAAGTTGTTATTCAGTTTTGCT

GCTTCTACTGATTTTGGTGCAACTAGGATGTGCTGCCTTCATATTCTTTGACAAAAATTGGAGAGATGAA
ATTCCTGGGGACAAAACAGGAAACTTTGATAAGATCTATGAGCTCCTGGAAGACAAGTGGGAAATCATCA
GATGGGTTGCATTAGGAGCTGTAATTTTTGAGGCTCTCCTTTTCTTGTTGGCGCTTGTGGTTCGTGCAGC
AAACAGACCTGTAGACTATGACAGTGATGATGAGTACATTGCTCCAAGGCAACAAATCCGACAACCGTTG
ATCAATAGGCCAGTTGCTCCTGCAACAGGTGTGCCTGTTGCTGGGACACTTGATCAACGACCAAGTCGAA
ATGATGCATGGAGTACACGAATGAGGGAAAAGTATGGGCTGGATACTTCGGAGTTCACATACAACCCATC
TGAGTCTCACAGGTTTCAGCAAGTTGCTCCTCAGCCAGCCGAAGAAAAGAGCCGCTGCACCATCATGTGA

Bch05G003940

ATGGAGGATATCAAGATGAAGATACGGACCTGCAGAAGAAAGAAGAAGAAAGTTGGACTAGAAGTTTGTC
AGCTGGAACGAAGAAGAAAGATGGTTGCCGACTGGAATGAAGAAGAAATGTTGGTTGGAACGACGCTGGC
TGTCGGCTGGACTGGAAGTTTGGAACGAAGAAGGCGAAGTTGGCTGCCGCCTTCCGAGTCGAACGAAGAA
GAAAGAAGTGCGTGA

Bch05G003950

ATGGTTTGTACTGCCGACGATTTACAAGAATGGAAAGACTTCCCTAAGGGTCTGAGGGTTCTTCTCCTTG
ATAGGGACAGTCGCTCTGCTACCGAGATAAGATCAAAACTTGAGGAAATGGAGTATGTTGTTTTTTCCTG
CTGTGATGAGAAGGAAGCTTTGTCGGCAATTTTGAACACACCGGGAAACTTCCATGTTGCAATTCTGGAG
GTGTGTGCAAGAAATTACGATGAAAGTTTTAAGTTACTTGGAACTTCCAAGGACTTGCCAATAATAATGA
CTTCAGATGTTCATTGCCTAAGTACCATGATGAAGTGCATTGCACTTGGTGCAGTTGAGTTCTTGCTGAA
ACCACTCTCTGAAGACAAACTCAGGAATATCTGGCAGCATGTCATTCACAAGGCATTTTCCAATACTTCA
AAGCCTGATGAAGACTCTGTAGCATCCTTGATGCAACTCCAATTAGAGAATGAAAACAAGAATGGAGTTT
CGGAAGATATGGAAGTTCTTTCTTGGATTCAGGATATTGTGTGGGAGGAACCAGAAGGAAGTGATAAGTC
TCAACTGATCATGGAAGCATCTAGGCAAGGTAGCTGGGAAAGCGGAGATCAAATGAACTGTTCAATAGAA
ACAGATTGCAGGGACAAAGATGTTCAGTCTAAATTCGTCGAAACTACTTCACATGATTTGGTTTGTGAAG
ACCCCATTCAGGAGGGCCAACCTCAATTATCTGACAAGAATAAATCTGGTGTCAAAAGTGATCCTTTAGC
TGCTGAAAACTCAATCCAAGGATCTGATGTGAACCATTCTGCTGGACCCAAAGCGAGGAAAACTAAGGTG
GACTGGAATCCACAGCTACATAGAAAATTTGTTCAGGCAGTTGAACAGTTAGGCATAGATCATGCAATTC
CTTCCAAAGTACTTGAGCTTATGAAAGTTGAAGGTTTGACAAGGCATAATGTTGCAAGTCATCTCCAGAA
GTACAGGATGCAAAAGAAACATGTAATGCAGAGAGAAGAAAATCCAAGGTGGTCACATTATCCAAGATGT
ACAATACAAACCAATCACTTGAAACCTATAATGGCTTACCCTTCTTCCTATCAACCAAACTGTGGAATAT
CAGTGTCTGCTGTTTATCCAACATGGAGACAGACCAATGGCCATCCACCTATTGTCCACACGTGGGGCCC
ACCTGGTTATAGCCATTGGCCGCAACAAGGAATTCAGCCATGGAATTCTTATGCAGGGGTGCGAGCTGAT
GCATGGGGTTGCCCTGTGATGCTGCCTTCTCACACTCCATATTTTTCATTTCCTCAGCATGCATCAGCAT
CACACGATATGCAGACAGTAAATAAGAGCTATGGCATGCCTCAGAGTTTATGTGATCTTCAACCAGATGA
AGAGGTGGTTGACAAGATTGTGAAAGAGGCAATGAGGAAGCCATGGTCACCGCTTCCATTAGGGCTCAAA
TCTCCTACAGAAAGTGTTCTCACAGAGCTTTCTAGGCAAGGAATCTCCACCGTCCCTCCTCAAATCAACG
GCTCCAGACCTCCCTGA

Bch05G003960

ATGTCTTCAACTCCGAAAAAGCGAACCAAAGTCAAACGTAATACGAACTCCGATGTCGGTTCTCGAGGCG
ATTCGTCTGTTTCATCTTCTACAATGTTGCTGAAGTCTATCAAGGAACCGCCTCGCGATTTCTTCCCCTC
GAAGGATGATCTTGCTGCGCTAATTACTGTACTCTTCATCGCCTGCTTGATTTTTGTGAGTTGTGACTTC
TTCGTATCTAGACTTGCAAGTCGCCAACCGAGGCCTTTCTGTGATACCGACGCTGATTCTTTGGATTTGC
TTTCTGATGCTTGTGAGCCTTGTCCAAGGCATGGAGAATGTCGTGATGGTAAGTTGAAGTGCCTTCATGG
TTATAGAAAGCATGGAAGGTTATGTATAGAAGATGGAGTAATCAATGAAGCAGTTAATAAACTTTCAGAA
TGGCTAGAATCTCACCTCTGTGAAGCAAATGTCAAGTTCTTATGCGATGGAATTGGGATAGTTTGGGTTA

AAGAGGATGATATATGGGATGATCTAGATGGTAAAGAGCTGGTGGAAAGTATTGGCTCTGACAACACCAC
TCTTACGTATGCAAAGAGTAAGGCGTTGGAAACTATTGGCGGGTTATTTCAGACGCGACAAAATTCTCTT
GGGATCAAGGAATTGAAATGTCCAGATCTGCTAGCTGAAAGTTACAAGCCTTTTACTTGCCGTATTCGTC
ACTGGGTTTTGCAGCATGCTTTCGCTGTTTTGCCAGTTTTCTTACTGCTTGTGGGATGCACATGGTTACT
TTGGAAACTTTACCGAAGACAATATATAACAAATAGAGCTGAAGATCTGTACAACCAGGTTTGTGAAATA
CTTGAGGAAAATGCTTTGATGTCAACAAGAAACAGTGGTCAATGTGAATCATGGGTTGTTGCTTCGAGGT
TACGTGATCACCTTCTTTTGCCACGAGAGAGGAAGAATCCTTTGTTATGGAGGAAGGTAGAGGAGTTGGT
TCAGGAAGACTCACGAATAGATCGTTACCCGAGACTAGTTAAGGGTGATGGAAAAGAAGTATGGGAATGG
CAAGTAGAAGGCTCTTTGAGCTCTTCAAAGGAAAAGAGACTGGCCACCAAATCCAATTCCGGGAAGGCAA
TGGGAGGAGACAATCAATTATTGGAACGAATTATGCTTAATCACAAAGTCAAATTTATGGATAAAAGAAA
TCCATCTGGCATGCATGTGATTGATGTTTTTGGGAATTTTCCTCTCATCACCAGGTCTGATCCGGATCTA
ATGGACTCATTTTGTGATGAGGTCATGTGGGAGAGAAGATCTGGGTATATGGTCAATGGCATCCAAGTCC
CTCTTGATAACTTTCCCATGGGAAATTGGACTGAGTAG

Bch05G003970

ATGTATGTCCTCCCAATGCCTTTGCTGTTTTTGCTGGCTCTGATAGTTATTTACTCTTTCCCGACTCAA
GAGGCAGCTCGGTGAATGCGGCGAAATCTTTAACGGGAGCTTCGGGGATTGGAAGCATTGCCATACCTGC
AATCTTGAAGCATGCTGATATGATAGGCTGGGGAGCACTGGCACTGGACTTCTCTTCCCTTTTAGTTCTT
GTGGTGGCCATTTTTTGTTATTTATGGATGAGTAAGGAGGAAGACTACAACATACTCCGAGTCTAA

Bch05G003980

ATGGGTCTCCTCTCATGGTGGAAAGGCCAATCTCCGCCATCGGACTCCAATTCCAAGCCACCGCCTCCTC
AGAAGAATGTCTCTCAACCTGCCGAAGTCCCCGGCCTGAATGGCGCCGTCGAAGTTCCCCGACCGGCTGC
CACTGTGACGGTCTTTGAGTTCGGCTCCGTTTCCTCTTCCTCCGACAAGGTCACTCTCGCCGGTTACTGC
CCCGTCTCCGATGACCTCGAGCCCTGCCGCTGGGAGATTCTGCCGGCAAGCGGCTCCGATGCGCCTCTTT
TTCGCGTCGTCTTCTGA

Bch05G003990

ATGGAGTTCCTGGACGAAGATGCGAAGCCCAGGTTCCTCTTCCACTCTCGCGCCAATCCATCTTCGGCGA
CGGAGCTCCAAACTGAATCTCAATCCAGCAAGCTCTTTGTCTCAATCACCGTCGTAATCTCTTCCATTTT
TCTCATTCTCTCAATTTTGTTCGTTCAATTCGAACCATTCAGATCCCTTCTCATCTGGCTTTCTCTCTCG
CTTCTTCTCGGCCCTTTCGCCCCAATTTCCCTCACCGGCGGCGACATTCGTGTCGGTCGAGGTCTGATTC
TCGAAATTCCCAACGAAGAACCCGAAGTAGAGGACGATGCCAAGAAGAAACCCGTTCAAAAGCGCTCAAA
ACCGCGTAGATCCGAGGAAATCGCGGTTGGCACAATCGAAGTTGCTGAAAAAGCCTCTTCAAAAATTGAG
AACAGGAATGGAGGAGTTCATCAGAGTAATAAAAATGGAGTCGATTTTGCAATCGAGGAGGCAGAATGGG
ACGAAGCGGAATTAGGGTTTTTGAAGAAGCAATTGACGAAACATCCTGTGGGGAAACCGAGACGGTGGGA
GATAATTGCAGAGGCGTTTGGTGGAAGGCATAAAGTGGAGAATGTGATTAAAATGGCTAAGGAAATGGGA
GAGAAGAAATTAGGCGATGAGGATTCGTATGCTCAATTTCTGAAGAAGAGGAAACCAATGGATAAGAGAA
TTGAGAACGTCAATGAAGAAGGTGCCACCGCTGTCGTCGACGGTCAGGTCGCCGGTTGGTCTTCTGGTGA
AGACATTGCATTGCTCAATGCTTTGAAAACGTTTCCAAAGGATTCGGCATTGAGATGGGAGAAAATTGCA
GCTGCTGTTCCAGGGAAGACGAAGGCAGCTTGTATGAAGAGAGTTGGGGAATTGAAAAGGGATTTTCGAA
ATTCTAAAGCTGCTAATGACATCTGA

qSC12

Bch12G020370

ATGGCGACCACAAATCCATTTGATTTGTTGGGTGATGACGACGCTGAGGACCCATCGCAGCTAATTGCCG
CCAAGCATGCGGCGGTTGCGGCGCCTAAGAAAGGTTCTGATCATCCTCAGGCTAAACAGGCGGCTGCGGC
AGCTAAACTGAACAAACCTGCTAATCTTCCTTCCAAGCCTCTTCCACCTGCTCAGGCTGTAAGGGAAGCA

AAGAATGAAGGTGGTCGTGGAGGTCGTGGTAGTGGGCGTGGTGGAGGTGGACGTGGATATGGGCGAGGGC
GTGGCAGTGGTGGCTTCAATCGTGAGTCTGCCAATAACGAGTACTCATTTAGTAGTAACCCTGAAGATGG
AGAGACTGGAAGGACAACAGAAAGACGTGGTGGATATGGTGGACCTCGTGGTCGTGGTGGCCGTCGTGGT
GGTTTTAATAATGGAGATGCTGTTGATGGTGAACACTCTCATGGTCTATTTGAACGCCACAGCGGAACTG
GCCGTGGAAATGAATTCAAGCGTGAAGGGTCTGGGCGTGGGAACTGGGGAAAGCCAACTGACGAATTTCC
TGAGGTTGCAGAGGGAGTCAATGAATCTGAGAAGAAGTTAGGTGATGAGAAGCCCGTTCATGAGGTTGAT
ACTGCAGGTGTCAACACAGAGAACTCTGCAAAAGAATCGGAGGAAAAAGAACCAGAAGACAAGGAAATGA
CTCTGGAGGAGTATGAAAAATTGCTGGAGGAGAAAAGGAAGACCCTTCTGGCACTAAAAACTGAGGAGAG
GAAGGTGGATCCTAAGGAATTTGCATCCTTGCAACAGCTTTCAAGCAAAAAGGAAAACCAGGACGTCTTT
ATTAAATTGGGATCTGATAAGGATAAGCGAAAAGAAACTGCAGATAAAGAGGAGAGAACTAAGAAGTCTT
TGAGTATTAATGAGTTCTTGAAGCCAGCAGAAGGGGAGAGACACTACACTCCAGGCGGTCGAGGTAGGGG
CCGGGGCCGTGGTGGAAGGGGAGGTTATGTTGGCAGCTCGGCGAGCAGCAATGTTCCAGCCCCGTCGATT
GAGGATCCGGGGCAGTTCCCAACCTTAGGTGCGAAATGA

Bch12G020380

ATGGCGACCGTTAATCCTTTTGATTTGTTGGGAGATGATGACGCTGAGGACCCGTCGCAGCTAATTGCGG
CTAATCAGGTGGCGGCGGTGGCGGCGCCGCCGTCTAAGAAAGGCCCTGCTCAGACGAAACCGGTGGCGAA
TAAGCCTGCTAGTCTTCCTTCCAAGCCCCTTCCACCTGCTCAGGCTGTAAGAGAATCAAGGAATGAAGGC
AGTCGTGGAGGTCGTGGCAGTGGACGGGGGGGAGGACGGGGATATGGGCGAGGTCGTGAGGGTGGTGGCT
TCAATCGTGACTTTGCCAATAATGAGAGCACTAATGCACCTGAAGATGGAGAGGGTGGAAAAACAGCGGA
AAGACGTGGATATGGTGGGCCTCGTGGTCGTGGTGGCCGTCGGGGTGGTTTCAATAATGGAGAGGCTGCT
GATGGGGAACGCCCTCGTAGAGCATTTGAACGCCACAGTGGAACTGGCCGTGGAAATGAATTTAAACGTG
AAGGGGCTGGTCGTGGAAATTGGGGAAGGTCAACTGACGAATTTGCTGAGGTGCCGGAGGAAGCTGTTAA
TGAAACTGAAAAGAATGTAGGTGACGAGAAGCCTATTCAAGAGGATGATACATCAGGGGTCAATACAGAG
AACCCTGCTAAAGAACCGGAGGAAGCAGTACCAGAAGACAAGGAAATGACACTGGAGGAGTATGAAAAGT
TGCTTGAGGATAAAAGGAAGGCCCTTCTAGCTCTAAAAACTGAGGAGAGGAAGGTGGATCCTAAAGAATT
TGCATCCATGCAACAACTTTCAAGCAAGAAGGACAACAACGAAATCTTTATTAAATTGGGATCCGAGAAG
GATAAGCGAAAAGAAATTGCAGATAAGGAGGAGAGAACTAAGAAGTCTTTGAGTATTAATGAGTTTTTGA
GGCCTGCGGAAGGAGAGCGACACTACGCCCCTGGTGGCCGAGGAAGGGGTCGTGGCCGTGGTTTAAGGGG
AGGATACAGTGGTAGCTCAATGAGCAATGTTGCAGCCCCATCCATTGAAGACCCGGGGCAGTTCCCGACC
TTAGGTGCGAAGTGA

Bch12G020390

ATGGCCATTCTTGCTCTTCCCCTGCTTGCCTCCTTCCTCAAACACCATACTTATCTCCTTTCCATTCGCT
CTCGGAAGTCACTTTCTTCTAGCTTCTGCAAGTCTCCATATCGCCGCCAATTCAACAAAACTTGTGCCTC
TGCCATTAGTCCTTCCACTACTCTACAGCATTCGTCCAGTGATTCCAAGACGTATGGCTCCAAGTCCTCG
GTTCTTACTTTTCAGCAAGCAATTCAGCGTCTCCAGGAATACTGGGCCTCTGTTGGATGTGCTGTGATGC
AATGCAGTAACACAGAGGTTGGTGCAGGAACCATGAATCCCTTGACATTCTTAAGAGTTCTTGGTCCAGA
ACCATGGAATGTTGCGTATGTGGAGCCTAGTATACGGCCAGATGATAGTCGCTATGGGGAAAATCCAAAT
AGACTCCAACGCCACACTCAGTTTCAGGTGATTTTGAAGCCTGATCCTGGAAATTCACAAGACCTTTTCA
TCCAAAGTCTCTCTGCTTTAGGGATTGATGTTGCAGCACATGATATACGTTTTGTAGAGGATAATTGGGA
GAGTCCGGTACTTGGCGCTTGGGGATTGGGATGGGAAATCTGGATGGATGGGATGGAGATTACACAATTC
ACTTACTTTCAGCAGGCTGGAAGTCAACAACTATTGCCTGTATCTGTTGAAATCACTTATGGTCTTGAAC
GTATTCTCATGTTACTGCAGGGTGTTAATCATTTTAAGAAAATTCAGTATGCTGATGGAATCACTTACGG
GGAGCTGTTCTTGGAGAACGAGAAGGAAATGAGTGCATATTATTTAGAACATGCCAATGTTCACCAGGTT
CAGAAACATTTCGACATCTTTGAGGAGGAAGCTCATTCTTTGCTTGCGTTAGGATTAGCAATCCCTGCGT

ATGATCAGGTTTTGAAGGCATCTCATGCTTTCAACATATTGGACTCAAGAGGCTTCATTGGGGTAACCGA
GCGTGCTCGTTATTTTGGTCGAATGCGAAGTTTAGCTCGTCACTGTGCGCAACTGTGGTTGAAAACCCGA
GAATCCCTAGGCCATCCACTTGGTGTTGTTTCTGATCCTGTTGATATTGTATGTCCCAAAGAACTTCTGG
ACGCAGCAGTCAAAAAGGTGCATGAAGATGCAAGGTGGTTTGTTCTTGAAATTGGAACCGAAGAAATACC
ACCTAAAGATGTTGTGGATGCAAGCCAACAACTCAAAATATATATGTTGCAGTTGCTTGAAAAGCATAGA
TTAAGCCATGGTAATGTGCAAGCTTTTGGCACACCTCGTCGGCTAGTGGTCACAGTTGAGAGTCTGTGTT
CTAAACAAGTAGAGAAAGAGATTGAGGTCAGAGGACCTCCTGTTTCAAAAGCTTTTGATGACCAAGGAAA
TCCTACAAAGGCTGCTGAGGGCTTTTGCCGCAGATACTCCATCTCACGTGAATCGTTGTATAGGAAGATC
GATGGAAAGACAGAATATCTATATGCTTCTGTAATGGAGTCTTCTCGGCTTGCTTTGGAGATTTTTTCTG
AAAATTTACCTGCCATTATAGCTAAAATATCCTTCCCAAAGTCAATGAGGTGGAATTCTCAGGTCGTGTT
TAGTAGGCCTATTCGTTGGATTTTGGCCCTCCATGGAGATGTAGTTGTTCCATTTTCATATGCTGGTGTT
TTAAGTGGGAACATATCCTATGGTCTTCGTAACACTTCTACAACTATTGTTGAGGTAGATAGTGCAGAAT
CATTTATGCGGACACTGAAGGATGCTAAAATTGATCTAGAAGTTAAGGACCGTAAAAGAAAAATCTTGGA
CCAGTCTTCCATATTGGCACAAAGTGTCAAAGGAAAAACTGTTATTCATGAGTCCTTGCTTGATGAGGTT
GTAAATCTTGTTGAGACTCCTGTTTCAATACTTGGAAAGTTCAATGATTCCTTCTTGGAGCTTCCTGAAG
ATCTTCTAACAATGGTTATGCAGAAGCATCAAAAGTATTTTTCAATTAGAAATCCTGATGGAAAGCTGAT
GCCATACTTCATTGCTGTAGCAAATGGAGAAATTGATGACAAAGTTGTAAGAAAAGGAAATGAAGCAGTA
CTTCGAGCTCGCTATGAAGATGCCAAGTTTTTCTATGAGACAGATACGAGTAAAAGGTTTTCTCAGTTTA
GAAATCAACTGAGTGGTATTCTGTTTCACGAAAAACTAGGATCGATGCTTGACAAGATGACACGTATGGA
AGCTATGGTTGTGAAACTAAGCCTCGCGATGGGAATTAGTCAAGATTTGCTTCAAATTATACAGGAAGCT
GCATCCCTTGCCATGTCGGATCTTGCTACTGCTGTTGTCACAGAATTTACTTCACTTGCAGGAATAATGG
CACGACATTATGCTTTGCGTGAGGGTTTTTCAGAGCAGATAGCAGAAGCCTTGTTTGAGATCACCCTTCC
CAGATTTTCTGGAGATATTCTGCCAAAGAGTGATGTTGGGATAGTTTTGGCTGTTGCTGACAGATTAGAT
AGCCTTGTTGGCTTATTTGCTGTTGGCTGTCAGCCTAGTTCTACAAGTGATCCATTTGGCTTGCGGAGAA
TTTCTTATGGTCTTGTCCAAATATTGGTGGAGAAGGATAAAAATCTTGATCTAGGGCGAGCACTGAGACT
TGCAGCTGATATCCAACCAATCAAAGTTGATACTAACGTAATTGATAATGTAATTCTATTTGTTACTCGA
AGGCTTGAACAATTTCTGGTGGATAAGGGTTTGAGTCCAGAAATAGTTCGTTCAGTTCTTGCAGAGCGTT
CAAATTTTCCTTGTCTGGCAGCAAAGACGGCTCATAAAATGAATGCTATGTCAAAAGGCGATCTCTTCCC
AAAGATTGTAGAGGCATATGCTCGTCCTACAAGAATCATTTCTGGGAAGGATGTTGACCCTGCTATGGAG
GTTGATGAGGCAGCCTTCGAATCGAATGAAGAAAGAGCTTTATGGAATACGTTTTTGTCAATTAAAAACG
AAGTTCACCCTGGCATTGAGGTTGATGAATTCTTTGAAATATCTTCAAAGCTCATTGAGCCACTTGAAGA
TTTCTTTGAGCGTGTATTTGTAATGGTGGAAAATGAAAAAATCCGTAAAAATCGGCTTGCTCTTCTCAAA
AAAATTGCTGATCTTCCAAGAGGCATAGCAGTGCTATCACTTCTATCAGGATTTTGA

Bch12G020400

ATGAGAACCTCAATCTCCATACGCCCATGCACATACTCTCCTCACTTTAATCAACCCTCCGCCACGGTCC
GCTCCAAGACGGCAAATCTCCGTCGTGTAAAGGTCATGCACTCCGCCGTTCGCCTCCGCATCAACGCCAT
GAAGAGGAACAACGTGGCTCATAAATGGACCGAGTACCAAGGCATTCAGAATTGGGATGGTTTGTTGGAC
CCATTGGATCACCATCTTCGAATGGAAATCCTTCGTTACGGTCGGTTCGTCGAAGCCGCCTACAACTCCT
TTCAATATGACGTTGATTCACCATTTTATGCAACATGTCGCCACTCAAAATCTTCGTTAATGAACCGAAC
TGGGTTGAGTCAGACCGGATATCGCCTCACCAAGTATTTACGAGCCACGTCCGGTCTAGAATTGCCACAT
TGGATCAAAAAGGCTGCCAACACAGCCATGAATCGATCTAGTTGGATCGGTTACGTGGCTGTGTGTGAGG
ACAAGAAAGAAATCGCCCGTCTCGGCCGTAGGGATATTGTGATCGCTTACAGAGGAACTGCCACTTGTTT
GGAGTGGATCGAGAATTTACGTGTTCCCCTAACTGAATTGCCTAATAATGACTCATATTCGGGTCAGGTT
GGGCCAAGGCCAATGGTGGAAACTGGGTTTCTGAGTCTGTATTCTTCTGGGACAGTGGGTTTGCCGAGTT

TGAAGCAAACAATACATGAGGAAGTTTCTAGATTACTCCACAGTTACGCCAGAGAGCCACTGAGTCTCAC
TATTACTGGCCATAGCCTCGGTGCTGCCTTAGCAATACTTACGGCATATGACATCAAAGTAACATTTGAA
CGAGTGCCACTTGTTACTGTCGTGTCTTTTGGTGGTCCTCGCGTAGGTAACAAAGACTTTCGAAGGAGTT
TGGATGAACAAGGTACAAAAGTCTTGCGCATCGTCAACTCCGACGACATTGTAACGAAGGTACCGGGATT
TGTGGTCGACGACGACAATGTGGAAGCACTGTCGTGCCCATGGTGGATCAAGCAATGTATGATGAATATG
CAGTCACAATATTGGTTGTACTCTGAAGTAGGGAAGGAACTGAAAGTGAACAACAAAAAGTCTTGGTATG
TTAATGGAGGAATAATGAATATGGGAATGCATCATGATTTAAAGACGTACCTCCATCTTGTTGAAGGCTT
TGTGAGCTCTAATTCTACGCTTGATGGTTCAACCAAGGCAGTTGCAGACGGTTACTTTTAA

Bch12G020410

ATGAAGACCAATTCACTCACTCCATCTCTTCCCCTCCAATTTCCATCGCCGATCAAACTTCCTCCGGTGA
CCGTTATCCGATTTCCATCTTCTCTAATTCCCAAAGCCATATCGATTCCCACTCCAAACACCACCCGCAT
ATCGATCAACGACGACGATTCCATCGCAGCCTTCTGGGACTACCAATTCCTCTTCGTCTCCCAGCGGACT
GAGACGACAGACCCCACCGTCCTCCGCCTAGTCGACGGCGCCATACCCCCCGATTTCCCCTCCGGCACGT
ACTACCTGGCGGGACCGGGCATGTTCTCCGACGACCATGGCTCCACAGTGCATCCACTCGACGGCCATGG
GTATTTAAGAGCGTTTGTTTTCGAGAGGAAGAAGAAGGAGGTGGTTTTCATGGCGAAGTACGTGAAAACG
GAGGCGAAAATGGAGGAGCATGATCTGGTGACCGACACGTGGCGGTTTACGCATAGAGGGCCGTTCTCGG
TTTTGAAGGGAGGGAAGAAATTGGGGAATACAAAAGTGATGAAAAATGTGGCTAATACGAGTGTGCTGCG
GTGGGGCGGCCGCCTGCTCTGCCTCTGGGAGGGCGGCGATCCGTACGAAATTCGGCCGGAGAATTTGGAT
ACGGTTGGCAAGTTTTCTGCTGTTCACGGTGGCTGTGATTGTGATTCGCCGTCCCACGGCGGACCTTACG
GTGCTGGGTTTTGGCGCTTTGCTGCGGAGCTTTTGAAGCCTGTGCTCTACGGAGTATTCAAGATGCCACC
AAAGAGATTGTTGTCTCATTACAAGCTTGATGCTCAAAGGAATAGACTTCTCATTATGTCATGCAATGCT
GAAGATATGCTTCTACCAACTAGCCATTTCACATTTTATGAATTTGACAGCAATTTCAAGTTATTACAGA
AAAAAGATTTGGTTATTGATGATCACTTGATGATCCATGATTGGGCTTTTACTGATACTCATTACATCCT
CTTTGCTAACCGAATCAAGCTCGATGTCATAGGTGCAATGGGTGCTGTTAGTGGGATATCTCCAATGATA
TCAGCATTAAGTGTAAACACAAGCAAACCCACTTCTCCAATTTATTTGATTCCAAGATTTGGTGAAGATT
CAAACAGAAATTTGAGAAAGGCTGTTGTTGAAGTTCCTTCAAGGCTTTGGCTGCTACACGTTGGCAATGC
CTTTGAAGTTATTCATGAAGATGGCAACTTGGACTTCCAAATTCATGCCTCTGCTTGCTCCTATCAATGG
TTCAATTTTAAAAATTTGTTTGGTTACAATTGGCAGACTGGAAAACTAGATCCTTCCATTATGAATTTGA
ATGAATCCAAAAGTGAGTTATTGCCTCGCCTAGTTAAGATATCCATCAGTTTAAATAAGAATGAGAAGTG
TGAAAAGTGCAGTGTGGAGCCATTGAACCAATGGACAAAATCCTCAGATTTTCCCGTGATAAACCCTAAA
TTCTCAGGGCTAAAACACAATTACTTATATGCTGCAACTTCCTCCGGTTATCGGCGATCGCTACCGTCGT
TTCCATTCGATATGATCGTGAAGCTCGACATGGTAATGAACACCGTCCGAACATGGTCTGCTGGTAATCG
AAGATTCGTCGGAGAACCGGTTTTTGTTCCCAAAGGAAATGAAGAAGATGATGGATATCTTCTTGTTGTT
GAGTATGCAGTATCAATACAAAGATGCTATCTTATTATTTTGGAGGCCCAAAGATTTGGAGAAGCTGATG
GAATTGTAGCAAGACTTGAGTGTAGGGAAACTTATTTTAGTGGTATGAATTTCCTATTGCTAGTTCACCA
CATGTACTATTAA

Bch12G020420

ATGGCCAAAAAAATGATTGCAGTGTTTCTTATGTGCATTGTGGTGATGGCTGCTTTGCAATTTACCACTG
CCAATGAAGAGGTGGCCAAATATGAGGCTAAATTTGATGCCAAATACAAGTCTTGCTATGAAACTTGTGA
GAAAGAATGTCTTGAAAAGGGCAATGGCCAAAGTTTTTGTGAAGTTAAATGTGATGAAGATTGTGATGAA
AAAGAAACTGCTGATAAGCTCCACATCAAGCCAGCACATTGA

Bch12G020430

ATGGCCAAGAAGATTATTGCAGTGTTTTTCATGTGCATTGTTGTGATGGCTGCTTTTCAATTTGCCACTG
CCAATGAAGAGGTGGAAAAATATGAGGCCAAATTTGAGGCCAAATACAAGTCTTGCTATGAAACTTGTGA

GAAGGAATGCCTTGAAGGAGGCAATGGCCAAAGCTTTTGTGAAGTTAAATGTGATGAGGATTGTGATGAA
AAAGAAACCGCTGATAAGCTACACATCGAATTGCATTGA

Bch12G020440

ATGGTTCGTGTTAGGGGAAGAGGCAGTCATAGGGTTACTGGAAGGGGGCGTGTTCCCGTAGAGCAGAATT
CTTCAGTTCAGGATCAAGATCCTCATGATGAGGATCCACAAGGTCAGGGTGGCCGTGAACCCGGACACAA
AGTAGAGCTCAGCACACGGCCATGCCGCCACAGACGAAGCCCACCCCCGTCACTCCAGCAGAGGTTATCG
ATTGGGCAGCCACTATTGGGGAAACAGTTACATCAGCTGTGTTGGACAAGGTAA

Bch12G020450

ATGGCCAAGAAGATTATTGCAATGTTTCTAATGTGTATTGTGGTGATGGGTGCTTTGCAATTTGGCACTG
CCAATGAAGAGGTGGACAAATATGAGGCCAAATTTGAGGCCAAATATAAGTCTTGTTATGAAACTTGTGA
GAAGGAATGTCTTCAAAATAGCAATGGCCAAAGTTTTTGTGAAGTTAAATGTGATGAAGATTGTGATGAA
AAGGAAGTTGCTGATAAGTTACACATCGGATTGCGTTGA

Bch12G020460

ATGGCCAAGAAGATGATTGCAGTGTTTTTTATGTGCATTGTGGTGATGGCTGCTTTGCAATTTGCCACTG
CCAATGAAGAGGTGGACAAATATGAGGCTAAATTTGATGCCAAATACAAGTCTTGCTATGAAACTTGTGA
GAAGGAATGTCTTGAAAATGGCAGTGGTCAAACCTTTTGTGAAGTTAAATGTGATGAAGATTGTGATGAA
AAAGAAAAAGCTGATAAGCTACACATCGAATTACATTGA

Bch12G020470

ATGTTGAATGCTAAGCGATTGTTTAAAAATAAAATCGTATTAATTGCGATTGATACTCAAATCAGGGAGA
CTAAATGGTTCTTAATCTTGAACTGTTTTCCAAATCTATGTTTTCCACAGGTTGATTGTGTCTGTTATCT
TCACAAAATCGTGATTGGTTTCTTTTTCCATTTGTTCTTGTGTGAAGGTCTACTTATATTCAGAGTATTT
CATTCTCTGAGGGTGAACATAAGAATCGGTGCTCATACGTCTGGGAGTAAAATGGTGAGGTATAAGGAAA
AAGCAAATAAAAAGCAGGCAGTTGAAGATGTGTCCCCGGTTGCTACAAACAAGTATGGTGTTCGTGTTTA
TGGAATGTCTATTACAAAGAAAGAGGTTGATGTTCTTCCCTCTTCGGCGAAACAAAGACGGTCTGGTGAA
AGCCAACCAGTGAAAACAAAAGGAAAAGAGAAATGTCTGTAG

Bch12G020480

ATGAAGCGAGACGGTGTCAATGTTGATTCCCACACTTTCGAGGTTCTTCTCGACGCGTTTATCAGGTCTG
GCAAATTCGATGCTGCCCTTGAAATTTTAGACCATATGGAAGATTTGGGAACTAGCTTGGATCTCAACAC
CTACAACTCTGTTCTTGTCGCTCTGCTCGGGAAAAACCAGGTGGGTTTGGCTTTGTCAATATTCTTTAAG
CTGTTAGATGGTTTTAGTAATGGAGGGCAAGAAGGTACTGCTGCAACTAGTTTTCCTTTCTTGCCTAATT
CACTTGCTTGTAATGAATTGCTGGTTGCTCTCAGGAAATCAGACATGAGGGTTGAGTTTAAAAGGGTTTT
TGACAAGCTTAGAGAAATTAGAACCTTTGAGTTTAATGTATGTGGTTATAATATATGTATTCATGCTTTT
GGATGTTGGGGTTATCTGGATACTTCTCTTGCCCTGTTCAAAGAAATGAAGCAAAAGAGCTTCGTTTTGG
AGTCGTTCGGTCCGGATTTGTGTACATATAATAGCCTTATTCACGTGCTCTGTTTGGTAGGGAAGGTTAA
GGATGCACTTATTGTGTGGGAGGAACTTAAAGGGTCAGGCCATGAGCCTGATGCCTTCACCTACCGTATC
ATAATTCAGGGTTGCTGTAAATCTTACCGAATGGATACCATTGTGTATAATTCTCTCCTCAACGGCCTAT
TTAAGGCTCGGAAAGTTACTGAAGCATGTCAACTTTTTGATAAAATGGTGCAAGAAGGTATAAGAGCTTC
TCCTTGGACGTACAATATTCTAATTGATGGATTGTTTAGGAATGGAAGAGCTGAAGCTAGCTACTCTTTA
TTCTGTGATATGAAGAAAAAGGGTCAATTTGTGGATGGTGTTACTTACAGCATCATTGTATTGCAACTGT
GTAAAGAGGGATTGCTTGAGGAAGCACTACAATTGGTTGATGAAATGGAAGCGAGAGGCTTCGTTGTCGA
TCTTATTACTGTAACATCTCTTTTAATTGCAATGCACAAGCAACGCCAGTGGGAAGGGTTAGAGAGGCTC
ATGAAGCACATTAGAGAAGGTAACTTGGTCCCCAATGTTCTCAAATGGAAGGCTAACATGGAAGATTCAA
TCAAATATCAGCAAAATAAAAGAAAAGACTACACATTCCTGTTCCCCCCAAAGGAGGATTTGAGTGAGAT
TATTAGTTCAAGAGCTTCTTCTGCTGCGGAAGTTAATGTTGACGATATTATCGAAAACACAGAAGAAACA

GATGCTGACAGTTGGTCATCATCCCCACATGTCGATCTTTTAGCTGATCTTGCAAAGTCTAATGGTGATT
TTTTGCAACCATTCTCTCTAGGTCAGGGGCGACGAATCCAAGCAAAAGGGGACAACTCATTCGATATCAA
TATGGTCAATACATTTTTGTCTATTTTTCTAGCAAAAGGAAAATTGAGCTTAGCTTGTAAGTTGTTTGAG
ATCTTCAGTGATATGGGTGTCAACCCGGTGAGGTATACCTACAATTCAATGCTGAGTTCATTTGTGAAGA
AGGGATACTTTCACCAGGCATGGGGTATATTTAACGAAATGGGCGAGAAGGTATGTCCAGCTGATATAGC
CACATACAATGTGATAATTCAAGGACTCGGGAAGATGGGTAGAGCGGATCTTGCAAGTTCTGTTCTGGAA
AAGCTAATGGAGCAGGGTGGCTATCTCGATATCGTAATGTACAACACATTGATTAATGCGCTGGGGAAGG
CAGGTCAAATGGATGACGTAAATAAGCTTTTTGAGCAGATGAGGAACAGTGGGATAAACCCAGATGTTGT
CACTTTTAATACACTTATCGAAGTTCATAGCAAAGCTGGTCGGTTTAATGATGCTTACAAATTCCTGAAA
ATGATGCTGGATTCAGGCTGTTCCCCGAATCATGTTACAGATACAACTTTGGATTTTCTGGGGAGAGAGA
TTGAGAAAGTGAGGTATGAAAAAGCTTCAATCATACGTGACAAAAACAGTTCTTGA

Bch12G020490

ATGCATTATCCAGCTCTCGCAAACACCCATTCAACCTTCTTCAAATGCACCCGATGGCAATTAGAAGAAA
CCTTAGACAAATTCTCCTGCCCTTTCCATTATTATTGTGACAGCATTTACCCTGGCGATTACCCTGCTGC
CATTGATTTACTCGTTCTTATCTTCACAGCAGCCACCTACATGTCGACCCTTTTGTTCATGCTAGCCGAC
ATGTCATTTCGTGGGAAATTTTGTTTAGATCAGCCAAAGAAGTTCCTTTTACCATCTGGGCCATTTTCAC
TTCCGATTTTTTTGTTTGTATTAGCCAAAGGCTATCGTATTAATACACTTTTTCCTCTCTTCTTAATGGG
TCCTCCAATTCTTCATATGGTTTACATTTCAGCTCTCACATTTGACAATGGAGCAGACAAGGATATTAAA
TATGTGTTCTTTGAAGCTTCAACAATGTCTGGGATTCTTCATGCCAGCTTGAATTTGGACTCTGTGATTC
TGCCTTATTACACAGGTTTGGATGCTTTGGTTGGGAGTGAGTTTTCTGGGGAATGCCCATCATGTGTTTG
TAGAAATGCCCCATTAGAAGTTGGGGGTAGATTTGTGTCTTATAGGGGTTGGTCGGGTACTACGTTTGTG
GTTGTGTGTGTTTTGTGTACGAGGATTGTGTGTCGGGTGGCAGGGGATAAGGTTATGAGAAAAGTTGTAG
TTTTGAAATGGCTGTTGGAAGGGTTGGGTTGGGTTTTGATAACATGGGATTGTGTTTATTTGAGTGCAAA
TTTGGGAGTGGAGAGAAGGGAATTGCAAGGTGTTGTTTATGGTTGTGTGTTTGGCTTGGTGTTTGTTCAT
ATCATCAAATTGCTGAGGAGATGGCATTTGATGTGTTCTGTGGGGAATCAAAACCAATTGGATCAAGTGT
AA
