# Supplementary material for: Quantitative Trait Loci Sequencing and Genetic Mapping Reveal Two Main Regulatory Genes for Stem Color in Wax Gourds
Source: Plants (Basel). 2024 Jun 29;13(13):1804. doi: 10.3390/plants13131804 (PMC11244448; doi:10.3390/plants13131804)
Supplement: Supplementary file 1 [file plants-13-01804-s001.zip › Supplementary Table S2.pdf]

Supplementary Table S2. Genotype identification of stem color in the germplasm resources of 55 wax gourd (qSC5)

Number	Variety Name	Stem color	Genotype
1	YF-27-1-1-1-1-2	Green	Green
2	YF-8-2-1-1	Green	Green
3	YF12-1-1-2	Green	Green
4	YO-8	Green	Green
5	MM1-1-1-1	Green	Green
6	GO-2-1-3	Green	Green
7	YO-6-2-1-2	Green	Green
8	Meng Shan	Green	Green
9	LI	Green	Green
10	YS-4-4-1	Green	Green
11	YF-22-1-1	Green	Green
12	TY-5-35-1	Green	Green
13	R44	Green	Green
14	R79	Green	Green
15	R68	Green	Green
16	YF-2-2-2	Green	Green
17	H-2	Green	Green
18	YF-2-1-2-2-2	Green	Green
19	YO -11-1-1	Green	Green
20	TL-1-2-2	Green	Green
21	YF-10-2-1-1	Green	Green
22	GH-1-2-3-1	Green	Green
23	C-32-1	Green	Green
24	Lv-5-2-1-1	Green	Green
25	yf-4-2-1	Green	Green
26	ymy-3-1-2-1	Green	Green
27	ys-1-1-6-1-1	Green	Green
28	MM-1-1-1-1	Green	Green
29	Huan Jiang	Green	Green
30	YF-104-1-2	Green	Green
31	yf-12-1-1-2	White	Green
32	YF-10-4-1	White	Green
33	YF-4-3-1	White	Green
34	YF-4-3-3	White	Green
35	YF-4-4-1	White	Green
36	DF-2-1	White	Green
37	YF-6-1-2	White	White
38	BC-3-3-1	White	White
39	YF-4-1-2	White	White
40	YF-4-1-1	White	White

41	Ymy-24-1-3-1	White	White
42	MM1-1-1-3	White	White
43	YF-F-1-1	White	White
44	YF-12-1-3	White	White
45	rl-10-14-1	White	White
46	HXK-4-1-1	Green	White
47	A-1	Green	White
48	YF-9-1-1-3-3	Green	White
49	DF-1-1	Green	White
50	YN-1-1	White	White
51	Ty-3-8-2	White	White
52	Rl-6-2-3	White	White
53	R80	White	White
54	Ym-3-3-3-1	White	White
55	YF-27-1-1-1-2	White	White

Genotype identification of stem color in the germplasm resources of 55 wax gourd (qS12)

Number	Variety Name	Stem color	Genotype
1	YF-27-1-1-1-1-2	Green	Green
2	YF-8-2-1-1	Green	Green
3	YF12-1-1-2	Green	Green
4	YO-8	Green	Green
5	MM1-1-1-1	Green	Green
6	GO-2-1-3	Green	Green
7	YO-6-2-1-2	Green	Green
8	Meng Shan	Green	Green
9	LI	Green	Green
10	YS-4-4-1	Green	Green
11	YF-22-1-1	Green	Green
12	TY-5-35-1	Green	Green
13	R44	Green	Green
14	R79	Green	Green
15	R68	Green	Green
16	YF-2-2-2	Green	Green
17	H-2	Green	Green
18	YF-2-1-2-2-2	Green	Green
19	YO -11-1-1	Green	Green
20	TL-1-2-2	Green	Green
21	YF-10-2-1-1	Green	Green
22	GH-1-2-3-1	Green	Green
23	C-32-1	Green	Green

24	Lv-5-2-1-1	Green	Green
25	yf-4-2-1	Green	Green
26	ymy-3-1-2-1	Green	Green
27	ys-1-1-6-1-1	Green	Green
28	MM-1-1-1-1	Green	Green
29	Huan Jiang	Green	Green
30	YF-104-1-2	Green	Green
31	yf-12-1-1-2	White	Green
32	YF-10-4-1	White	Green
33	YF-4-3-1	White	Green
34	YF-4-3-3	White	Green
35	YF-4-4-1	White	Green
36	DF-2-1	White	Green
37	YF-6-1-2	White	Green
38	BC-3-3-1	White	Green
39	YF-4-1-2	White	Green
40	YF-4-1-1	White	Green
41	Ymy-24-1-3-1	White	Green
42	MM1-1-1-3	White	Green
43	YF-F-1-1	White	Green
44	YF-12-1-3	White	Green
45	rl-10-14-1	White	Green
46	HXK-4-1-1	Green	White
47	A-1	Green	White
48	YF-9-1-1-3-3	Green	White
49	DF-1-1	Green	White
50	YN-1-1	White	White
51	Ty-3-8-2	White	White
52	Rl-6-2-3	White	Green
53	R80	White	Green
54	Ym-3-3-3-1	White	Green
55	YF-27-1-1-1-2	White	Green
