# Supplementary figures and images for: Quantitative Trait Loci Sequencing and Genetic Mapping Reveal Two Main Regulatory Genes for Stem Color in Wax Gourds
Source: Plants (Basel). 2024 Jun 29;13(13):1804. doi: 10.3390/plants13131804 (PMC11244448; doi:10.3390/plants13131804)

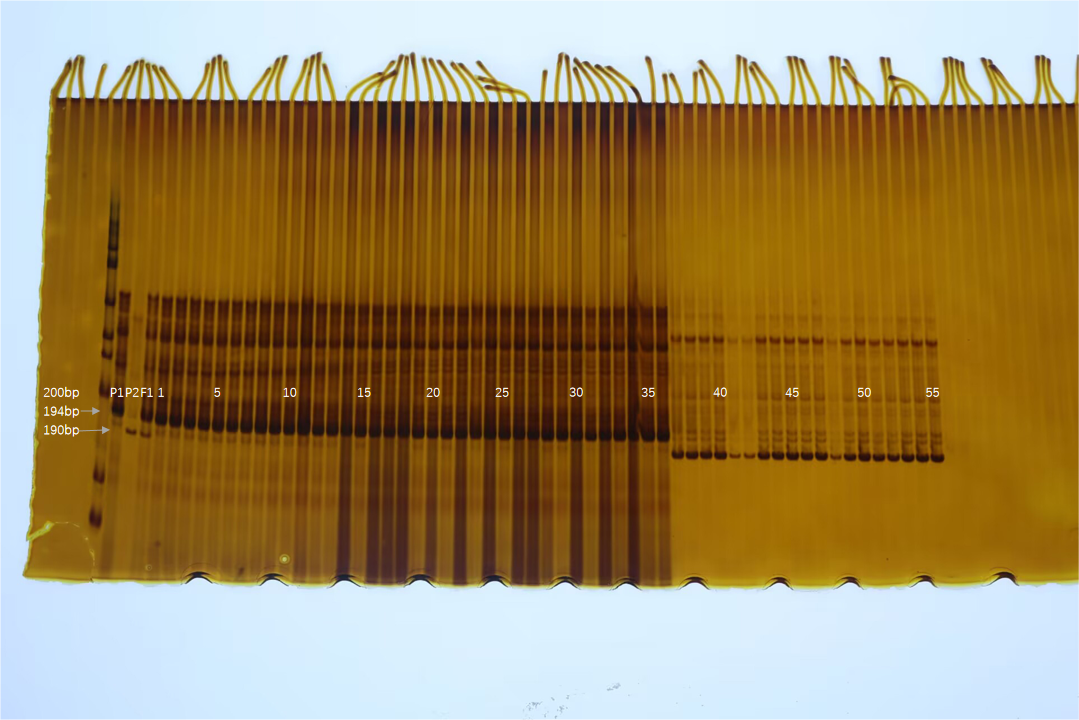

Supplement: Supplementary file 1 [file plants-13-01804-s001.zip › Supplementary Figure S1.tiff]
